# Ontogenetic changes in cortical bone vascular microstructure in the domestic duck (*Anas platyrhynchos*) and ring‐necked pheasant (*Phasianus colchicus*)

**DOI:** 10.1111/joa.13741

**Published:** 2022-08-24

**Authors:** Katherine A. Williams, Neil J. Gostling, Richard O. C. Oreffo, Philipp Schneider

**Affiliations:** ^1^ School of Biological Sciences, Faculty of Science and Health University of Portsmouth Portsmouth UK; ^2^ Faculty of Engineering and Physical Sciences University of Southampton Southampton UK; ^3^ Institute for Life Sciences University of Southampton Southampton UK; ^4^ School of Biological Sciences, Faculty of Environmental and Life Sciences University of Southampton Southampton UK; ^5^ Bone and Joint Research Group, Centre for Human Development, Stem Cells and Regeneration, Institute of Developmental Sciences University of Southampton Southampton UK; ^6^ High‐Performance Vision Systems, Center for Vision, Automation & Control AIT Austrian Institute of Technology Vienna Austria

**Keywords:** avian, bird, bone, development, histology, microstructure, palaeontology, vasculature

## Abstract

Age‐related changes in bone microstructure can inform our understanding the biology of both extant and fossil birds, but to date, histological work in birds, and particularly work using high‐resolution 3D imaging, has largely been restricted to limited growth stages. We used minimally destructive synchrotron radiation‐based X‐ray computed tomography to visualise and measure key morphological and histological traits in 3D across development in the domestic duck and ring‐necked pheasant. We use these measurements to build on the database of key reference material for interpreting bone histology. We found that growth patterns differed between the two species, with the ducks showing rapid growth in their lower limbs and early lower limb maturation, while pheasants grew more slowly, reflecting their later age at maturity. In the pheasant, both walking and flight occur early and their upper and lower limbs grew at similar rates. In the duck, flight and wing development are delayed until the bird is almost at full body mass. Through juvenile development, the second moment of area for the duck wing was low but increased rapidly towards the age of flight, at which point it became significantly greater than that of the lower limb, or the pheasant. On a microstructural level, both cortical porosity and canal diameter were related to cortical bone deposition rate. In terms of orientation, vascular canals in the bone cortex were more laminar in the humerus and femur compared with the tibiotarsus, and laminarity increased through juvenile development in the humerus, but not the tibiotarsus, possibly reflecting torsional vs compressive loading. These age‐related changes in cortical bone vascular microstructure of the domestic duck and pheasant will help understanding the biology of both extant and fossil birds, including age estimation, growth rate and growth patterns, and limb function.

## Graphical abstract

We used minimally destructive synchrotron radiation‐based X‐ray computed tomography to visualise and measure macrostructural traits and intracortical microvasculature in 3D across development in the domestic duck and ring‐necked pheasant. These age‐related changes in cortical bone vascular microstructure of the domestic duck and pheasant will help understanding the biology of both extant and fossil birds, including age estimation, growth rate and growth patterns, and limb function.

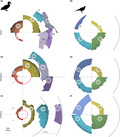

## 1 INTRODUCTION

Understanding bone microstructure in animals is important for answering biological questions related to ontogeny (Scannella & Horner, 2010), biomechanics (Pratt & Cooper, 2018), and phylogeny (Gao et al., 2012; Scannella & Horner, 2010), in both extant and extinct species. In particular, bone histology is the best way that we have to estimate traits such as growth rate in fossils, and has been linked to locomotory traits such as flight (de Margerie, 2002; Simons & O'connor, 2012). While historically, histology has relied on production of thin sections, minimally destructive X‐ray CT methods are becoming increasingly widespread, enabling the characterisation of 3D traits such as vascular networks (Pratt & Cooper, 2017; Pratt et al., 2018). However, the number of comparative studies in extant species using these techniques is, to date, limited and without extant comparative datasets, interpretation of fossil material is challenging.

Even based on 2D sections, questions remain about functional links between the structures observed in histological sections and the biology of the animal and the depositional processes that produce bone tissue, including how the organisation of the microvascular network in cortical bone tissue correlates with traits such as growth rate (de Margerie et al., 2002, 2004; Kuehn et al., 2019; Pratt & Cooper, 2018) and mechanical function of the bone (de Margerie, 2002, 2005). While, due to developmental plasticity, it is unlikely that absolute relationships between e.g. absolute bone deposition rate and exact bone structure that can be directly extrapolated to fossils will ever be found (Starck & Chinsamy, 2002), correlations exist between histological traits and biology.

Both growth rate and bone loading change with age, but developmental changes are difficult to study in fossils where absolute ages are unknown and locomotory behaviours are uncertain. Thus, studying how key traits change through ontogeny and correlate with behavioural transitions such as the onset of flight in birds is an approach that can help us to understand development and behaviour in fossil birds.

Due to the way in which bone is deposited, its structure undergoes substantial changes through ontogeny. In very young bone, most of the bone tissue is of the woven type, with irregular arrangement of collagen fibres and a porous structure (Marotti, 2010; Palumbo et al., 2002). In this type of bone, the tissue is quickly laid down but mechanically weak. The spaces are gradually infilled or replaced as the bone ages (Stein & Werner, 2013) and the bone becomes stronger as the strength of cortical bone tissue is strongly inversely related to its porosity (Cooper et al., 2016). In fibrolamellar bone, the large spaces in the initially deposited bone, containing vascular canals, are infilled by concentric layers of bone, forming primary osteons. Blood vessels and nerves remain in the middle of the osteons providing oxygen throughout the tissue. In adult bone, very little woven bone is present (Stein & Werner, 2013).

Although the same processes occur to produce fibrolamellar bone in most birds, there is a wide variation in growth rates, life histories and lifestyles between species and bone growth is modified to adapt to these differences. Even within a species bone development can be plastic, reflecting the actual availability of food and behaviour of that animal (Starck & Chinsamy, 2002). These variations are reflected in the structure of the bone. For example, although all bone is deposited around blood vessels, how those vessels are organised is variable, from the density of the vascular network to the main orientations of the vessels (longitudinal, laminar, radial or oblique). The organisation of vasculature that runs through cortical bone has been associated with bone growth rate (de Margerie et al., 2002, 2004; Kuehn et al., 2019; Pratt & Cooper, 2018) and bone mechanics (de Margerie, 2002, 2005), both of which might change through ontogeny. For example, radially oriented canals (like the spokes of a bike wheel) have been correlated with more rapid tissue deposition (Pratt & Cooper, 2018), while longitudinal (along the length of the bone) and laminar (running circumferentially around the bone) canals have been correlated with slower tissue deposition, although evidence for this relationship is mixed (de Margerie et al., 2002; Pratt & Cooper, 2018).

In terms of mechanical requirements, a laminar canal orientation is hypothesized to help a bone to resist torsion (de Margerie, 2002; de Margerie et al., 2002, 2005), possibly through associated collagen fibre orientations(de Margerie et al., 2002) which may be most important in the wing bones of flighted birds (Norberg & Aldrin, 2010). It is probable that the requirement for torsional resistance in flight‐related bones such as the humerus increases through life, as few birds can fly from hatching. Therefore, it may be predicted that the vascular arrangement in these bones would become more laminar with age, but this has not yet been tested (Pratt & Cooper, 2018).

In addition to understanding relationships between form and function, understanding how bone microstructure changes with age can improve estimates of the developmental age in an unknown sample (e.g. a fossil). Developmental age of specimens is crucial for accurate taxonomy and for understanding the evolution of particular traits. In several dinosaur species, the validity of certain species have been strongly debated for this very reason (Maiorino et al., 2013; Scannella & Horner, 2010), identifying juveniles is necessary for studying e.g. trends in body size (Griffin & Nesbitt, 2020), and identification of a sub‐adult Early Cretaceous bird has led to re‐identification of other specimens (Gao et al., 2012). Even in the most iconic bird species *Archaeopteryx lithographica*, several different age stages have been identified (Rauhut et al., 2018). Adding to the comparative library of extant studies will contribute to a more robust interpretation of fossil specimens.

This study characterises how microvasculature within cortical bone changes with age, growth rate and limb function, in two model species, the domestic duck (*Anas platyrhynchos*) and ring‐necked pheasant (*Phasianus colchicus*). 3D data on macrostructure and diaphyseal microstructure of avian bones were collected throughout development in a growth series of each species using synchrotron radiation (SR)‐based computed tomography (CT). These species were chosen as they are closely related (both within Galloanserae), have similar body masses and are both precocial, minimising the influence that phylogenetics or body mass might have on differences between the two species. However, they have some key differences in developmental patterns, in particular the age of first flight and flight capability. Therefore this study aimed to capture these key transitions in development and function and characterise the associated changes in bone microvasculature, as well as describing the structures observed at different age stages.

## 2 MATERIALS AND METHODS

### 2.1 Materials

Samples of *A. platyrhynchos* (commercially produced strains of a Mallard/Pekin cross known as Cherry Valley) were donated by Warawee Duck Farm, Southampton, UK across the full developmental range from 1 day old to 2 years old. Samples of juvenile *Phasianus colchis* were donated by Blackmore Game Farm, across the juvenile age range of 15–42 days. This provided an ontogenetic series for each species that could then be used to create growth curves and study microstructural changes (detailed in Methods). The samples were individuals that were naturally deceased throughout development. No live animals were used, no animals were killed for this study, and ethics were approved by the University of Southampton (ERGO number 27443). In order to study growth and remodelling separately, duck samples were split into three age classes, defined as: juvenile (1–42 days of age, when the animal is growing to full size), sub‐adult (42 days to 6 months, at which time the animal is approximately adult body mass but has not yet reached maturity, i.e., has not yet begun laying eggs), and adult (older than 6 months, i.e., has begun laying eggs).

For the ducks, the ages were provided for one 1‐day‐old, one 6‐week‐old, one 3‐month‐old and three 2‐year‐old individuals, and the body masses of all individuals were recorded. The individuals of unknown age were all larger than the 1‐day‐old and smaller than the 6‐week‐old individual. Based on the growth curves for ducks presented by Montes et al. (2005), the individuals within this age range are expected to fall within the near linear portion of the growth curve for ducks (Montes et al., 2005). Thus, the ages of individuals between 1‐day‐old and 6‐week‐old were linearly interpolated using the recorded body masses assuming linear growth in body mass between 1 day and 6 weeks. For pheasants, all ages were provided. In pheasants, juvenile development is more prolonged, and at 100 days they are still growing (Montes et al., 2005). Therefore, all pheasant samples, including those at 42 days were classed as juvenile.

To create a complete growth curve for pheasants for comparison with ducks, adult bone measures were obtained from literature. Namely, adult maximum bone length for the pheasant tibiotarsus and humerus was obtained as the mean value for the relevant pheasant bone length measurements recorded in (Watson & Ledogar, 2019). This length was used to calibrate the scale drawings in (Cohen & Serjeantson, 1996), from which the bone diameter at the mid‐diaphysis was derived using the ‘Measure’ tool in the Fiji distribution of the free, open‐source image processing package ImageJ 1.52 (Doube et al., 2010; Rueden et al., 2017).

### 2.2 Sample preparation

The humerus, femur and tibiotarsus were harvested, and the maximum lengths and minimum diameter at the mid diaphysis were measured using digital callipers. For the smallest bones (less than 2 mm diameter), the precision of the diameter measurement was increased by measuring the minimum diameter from the SR CT scan using the ‘measure’ tool in Fiji.

Samples were prepared to ensure the whole width fit into the lateral field of view (FOV) while imaging by SR CT (approximately 2 mm across). For smaller bones, where the diameter was less than 2 mm, the cortex was left intact, but midshaft sections of larger samples were cut lengthways using a slow‐speed saw (Buehler Isomet, Esslingen, Germany) to produce quadrant sections of the long bones. The same quadrant was used from each bone for imaging (antero‐ventral for the humerus, antero‐medial for the tibiotarsus). Although it is possible to image local regions of an entire larger specimen using local CT (i.e., the sample is bigger than the lateral FOV of the detector) at higher spatial resolutions, artefacts appear in the SR CT reconstructions due to the missing image information outside the FOV that is covered by the imaging setup. Therefore the material was prepared to fit within the FOV in order to optimise image quality. Samples were fixed using 4% paraformaldehyde (PFA) for at least 48 h and then transferred to 70% ethanol.

### 2.3 SR CT imaging

Images were collected over two beam times, one awarded at TOMCAT beamline of the Swiss Light Source (Paul Scherrer Institut, Villigen, Switzerland) and the other at I13‐2 of Diamond Light Source (Harwell Science and Innovation Campus). Since the equipment is not directly equivalent, scans could not be made at identical settings, however, the contrast between the sample and background was strong (bone vs air/water) and objects of interest were significantly larger than the pixel resolution, so slight differences in imaging setup should not have had a significant impact on the results. To minimise any impact of variation, images were binned by pixel averaging to the same voxel sizes.

For most samples, the mid‐diaphysis of the samples were imaged by SR CT at TOMCAT beamline of the Swiss Light Source (Paul Scherrer Institut) using a monochromatic beam at an X‐ray energy of 21 keV. The voxel size was set to 1.6 μm. 180 ms exposure per projection was chosen for 1501 projections over a 180° rotation at a sample‐to‐detector distance of 12 mm. Each SR CT scan took approximately 6 min, which were reconstructed using *gridrec* (Marone & Stampanoni, 2012). The final reconstructed SR CT volume was 2560 × 2560 × 1982 voxels^3^ or 4.1 × 4.1 × 3.2 mm^3^ and saved as 16‐bit TIFF stacks.

For pheasant scans and a small number of duck sample scans (see Tables S1 and S2), the mid‐diaphysis of the samples were scanned at beamline I13‐2 of Diamond Light Source (Harwell Science and Innovation Campus), using a pink beam with an average X‐ray energy of 20 keV. The voxel size was set to 0.8 μm. 100 ms exposure per projection was chosen for 4001 projections over a 180° rotation at a sample‐to‐detector distance of 20 mm. SR CT scans were reconstructed using standard filtered backprojection, then binned twice to a final voxel size of 1.6 μm to match the voxel size of datasets collected at the Swiss Light Source. In addition, the stack size was reduced from 1079 slices to 1000 slices by removing slices at the top and bottom of the stack that were of lower image quality compared to the rest of the SR CT stack. The final SR CT volume was 1280 × 1284 × 1000 voxels^3^ or 2.0 × 2.1 × 1.6 mm^3^.

### 2.4 Image segmentation

**FIGURE 1:**
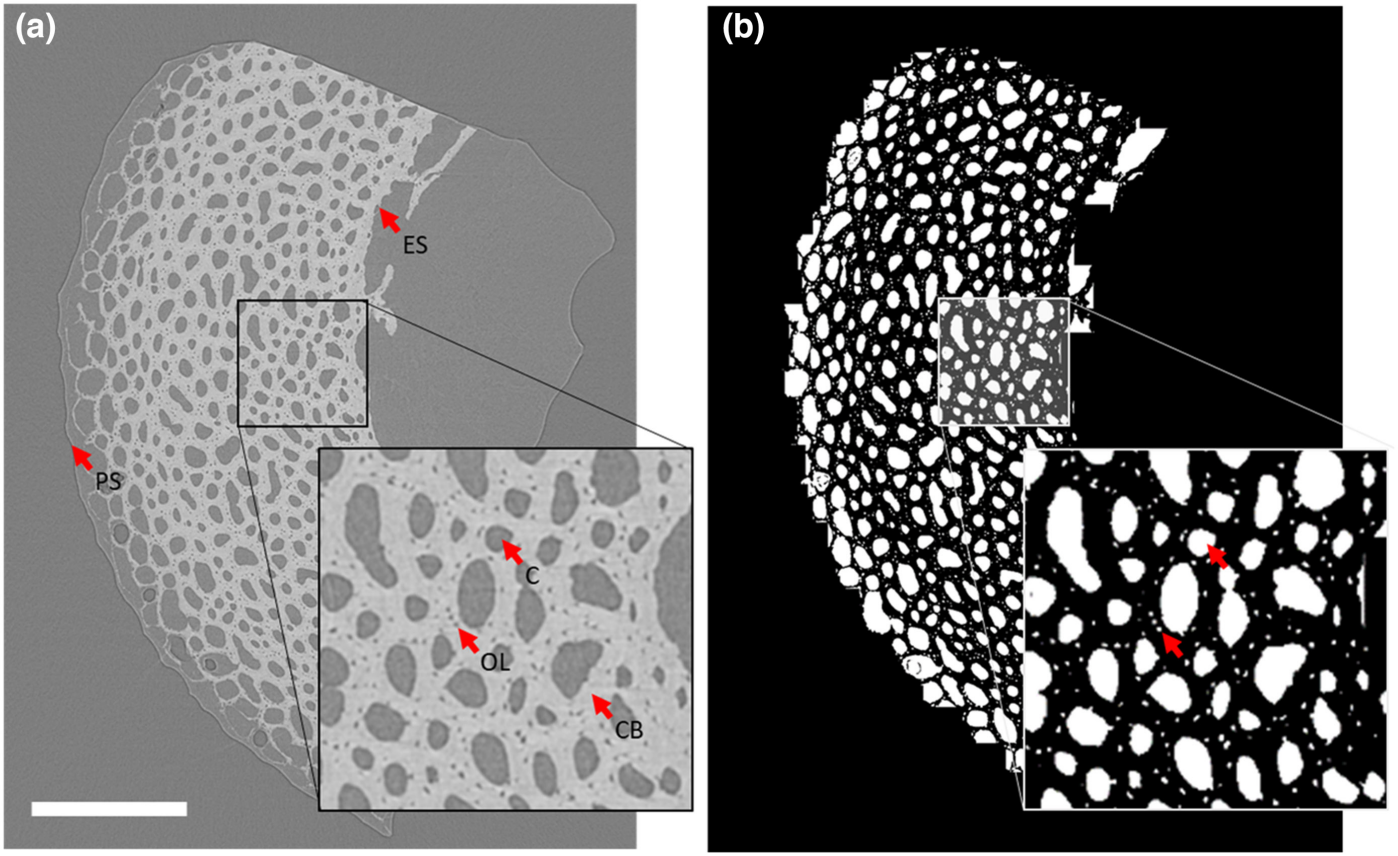
Pre‐ and post‐segmentation images for juvenile duck bone (age 15 days). (a) Greyscale slice from SR CT stack. (b) Segmented pores showing both vascular canals and osteocyte lacunae. PS, periosteal surface; ES, endosteal surface; OL, osteocyte lacuna; CB, cortical bone; C, canal. Scale bar = 500 μm.

For samples less than 2 mm in diameter, where the entire bone diameter was imaged (see above), the image stack was cropped to the quadrant of interest so that the analysed region was equivalent across all samples. Then, the vascular canals were segmented using a custom workflow in Fiji. The pores or canals found within the bounds of the mineralised tissue that were larger than 1000 μm^3^ (the volume below which we define pores to be osteocyte lacunae; Williams et al., 2020) were isolated from the 3D datasets (Figure 1) using the following process. First, images were binarised using a minimum cross‐entropy thresholding algorithm (Li & Tam, 1998) to identify the mineralised regions of bone tissue. The canals within the mineralised regions were filled using a series of dilations followed by erosions and the solid bone image was used as a mask to separate the pores within the bone from the background. Medullary bone found inside adult femura was removed manually. The custom workflow used is described in further detail in Williams et al. (2020), and specific details can be found in the Supplementary Methods and Figure S1. As this work focused on vascular canals and the spatial resolution was not high enough to accurately measure the osteocyte lacunae (Williams et al., 2020), pores smaller than 1000 μm^3^ were excluded using the ‘Particle analysis’ tool in BoneJ (Doube et al., 2010), a plugin for ImageJ designed for quantifying bone structure.

### 2.5 Data analysis

Segmented SR CT scans were analysed using Fiji. The ‘Particle Analysis’ tool in BoneJ was used to measure volumes of the canal network and the total cortical bone volume (defined as the mask used in the ‘Image segmentation’ section). Porosity was defined as the volume of the canal network divided by the volume of the cortical bone. Canal diameter was estimated using the ‘Thickness’ tool in BoneJ, which uses sphere fitting to derive the mean thickness as the arithmetic mean of the local thicknesses taken over all points in the structure. Cortical thickness was estimated from the solid bone mask described in the ‘Image segmentation’ section above using the ‘Thickness’ tool in BoneJ.

We were interested in assessing the bending stiffness of the bones. Assuming that mineralised regions of bone have similar mechanical properties, we compute changes in the second‐moment area, which is related to bending stiffness. The second moment of area was calculated as:

$$I=\frac{\pi }{4}\left(r_{2}^{4}-r_{1}^{4}\right)$$

where $r_{2}$ is the radius of the midshaft of the bone ($\frac{\text{diameter}}{2}$) and $r_{1}$ is the internal radius (subtract cortical thickness from $r_{2}$). This measure is a simplification and does not take into account the porosity of the bone because pore measurements and locations were not available across the entire bone, only one quadrant. Therefore the second moment of area measurements are likely to be overestimations, particularly in the most porous bone.

**FIGURE 2:**
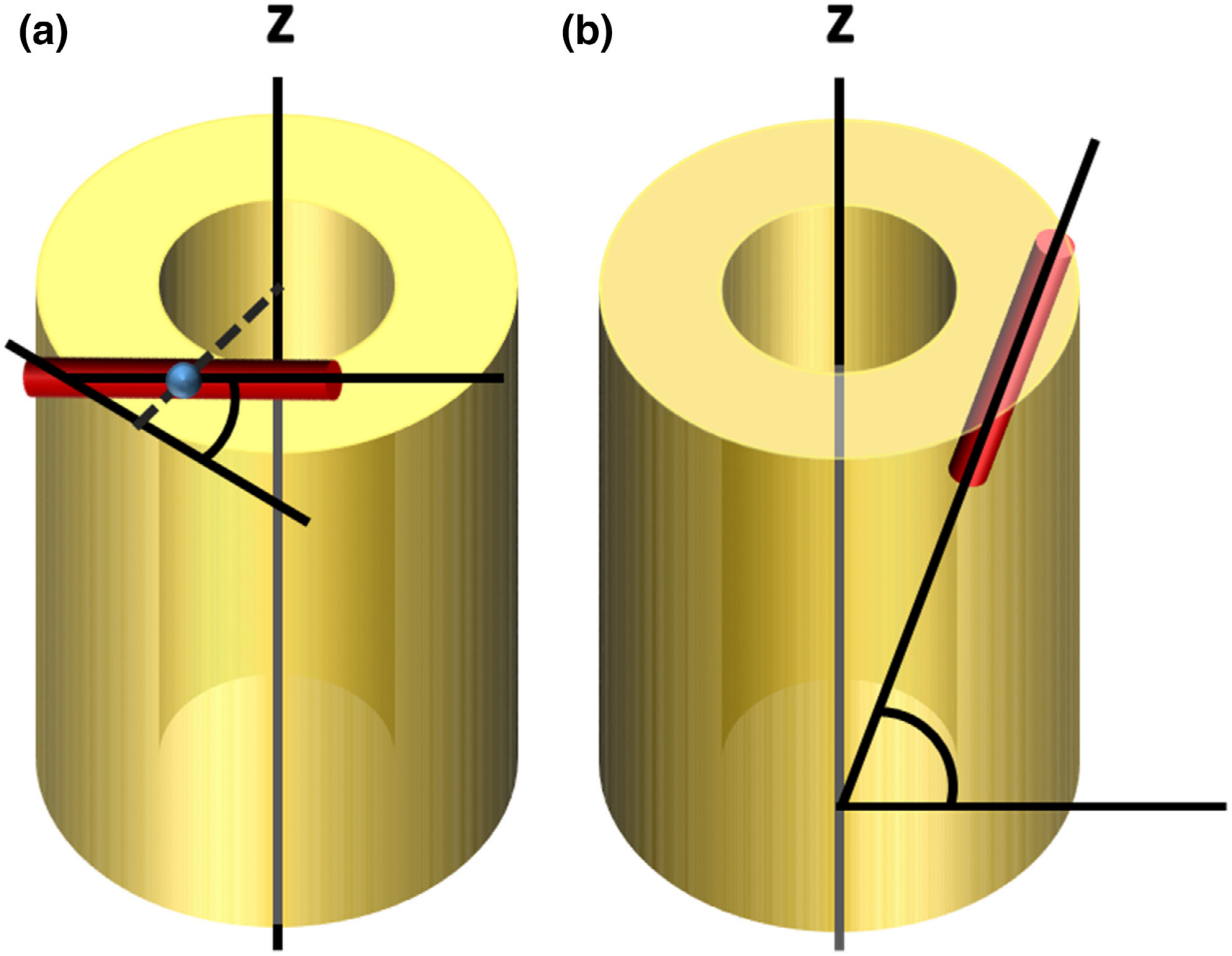
Radial and longitudinal angle measurements input into angle indices. (a) Radial angle is defined as the angle the canal deviates from the nearest tangent to the bone surface (b) longitudinal angle is defined as the angle of the canal relative to the long axis of the bone (*z*), where an angle parallel to the long axis of the bone corresponds to 90 degrees. Red cylinders represent vascular canals. The blue sphere indicates the midpoint of the canal.

Major orientations of the canal network have been related to bone growth rate and mechanical properties, so we were interested to test whether the orientations changed through development to reflect either changing growth rates or changing use of the bones. Canal orientation was estimated using the implementation of Pratt's 2017 method (Pratt & Cooper, 2017) as described in Williams et al. (2020). Segmented canal networks were thinned to single pixel lines (skeletonised) using the ‘Skeletonize 2D/3D’ tool within BoneJ, and the network was analysed using the ‘Analyze Skeleton’ tool within the same plugin. The branch points of the skeleton were used to define individual canals as straight segments. Using a custom Python script (Williams et al., 2020), the orientation of each segment was characterised, by calculating a radial angle (angle the canal deviates from the nearest tangent to the bone surface Figure 2a) and a longitudinal angle (angle relative to the long axis of the bone, where an angle parallel to the long axis of the bone corresponds to 90°, indicating how much the canal deviates from the main longitudinal axis of the bone; Figure 2b). These two angles were then used to calculate a radial index, a laminar index and a longitudinal index, describing the proportion of canals in the network with those specific orientations, following De Boef and Larsson (2007) (Table 1). Angles not falling into the longitudinal, radial or laminar categories are classified as oblique. The indices were weighted by canal length to reduce the undue influence of short canal segments.

**TABLE 1:** Angle categories for longitudinal, radial and laminar indices for assessing main vascular organisations in bone, following De Boef and Larsson (2007). Angles not falling into the longitudinal, radial or laminar categories were classified as oblique.

Orientation category	Longitudinal angle (°)	Radial angle (°)
Longitudinal	67.5–90.0	0.0–90.0
Radial	0.0–67.5	67.5–90.0
Laminar	0.0–67.5	0.0–22.5

### 2.6 Statistics

Statistical analyses were carried out in Origin 2019b (OriginLab Corporation) and RStudio (Version 1.2.1335; RStudio, Inc). Pearson's correlation coefficient (Origin) was used to characterise correlations where the dependent variables were unbounded (canal diameter, cortical thickness and mid‐diaphyseal diameter during juvenile growth). Where dependent variables were proportions (porosity, canal orientation indices), a beta regression with a logit link was implemented using the *betareg* package in RStudio. *p*‐values were obtained using the ‘lrtest’ function in the ‘lmtest’ package, and the summary function in ‘betareg’ used to produce a pseudo R‐squared value, the squared correlation of linear predictor and link‐transformed response. Full model outputs can be found in Table S3. In Origin, ANOVA was used to test for differences between means, and Tukey's multiple comparison test was used to test for pairwise differences. In Origin, growth curves were fitted using a sigmoidal Gompertz model, a model that is commonly used to study growth (Vincenzi et al., 2020; Winsor, 1932). The growth rate was calculated as the first differential of the Gompertz models fitted.

## 3 RESULTS

**FIGURE 3:**
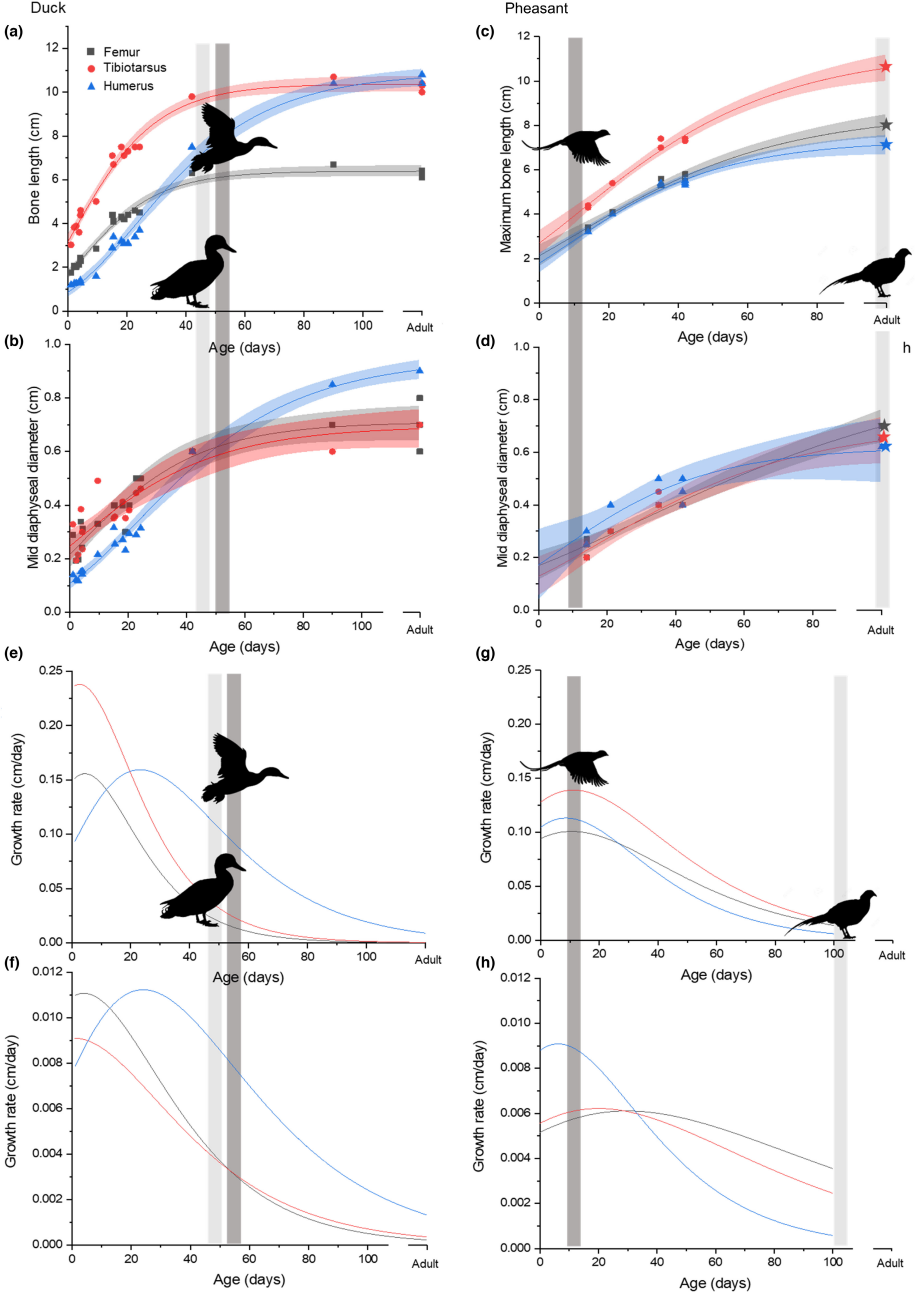
Growth curves for the femur, tibiotarsus and humerus in the duck (*Anas platyrhynchos*) and pheasant (*Phasianus colchicus*). Stars indicate mean bone length values from (Watson & Ledogar, 2019)and bone diameter estimates based on 34 (Cohen & Serjeantson, 1996). (a and c) maximum bone length, (b and d) minimum mid‐diaphyseal diameter. Lines were fitted using a Gompertz model (Winsor, 1932) and shaded areas indicate 95% confidence intervals. Light grey bars and standing silhouettes mark approximate age at full body mass, dark grey bars and flying silhouettes mark estimated appearance of first flight (Montes et al., 2005). (e‐h) The growth rate calculated as the first differential of the Gompertz fit. (e and g) Length rate, (f and h) radial growth rate.

Bone length and diameter were used to assess growth patterns through growth curves, both in terms of absolute size and growth rate in both dimensions (Figure 3). In the duck, the tibiotarsus had a higher maximum longitudinal growth rate than the femur (2.38 and 1.56 mm/day respectively) and lower radial growth rate than the femur (0.091 and 0.111 mm/day respectively). The humerus grew more slowly at early stages of development than the lower limb and its maximum growth rate was later than for the lower limb bones (Figure 3a,e). It had a similar maximum longitudinal and radial growth rates to the femur (1.59 and 0.0112 mm/day respectively), but reached its maximum longitudinal growth rate at 24 days, 10 days later than the femur and 13 days later than the tibiotarsus. Therefore, from a relatively small size early in growth, the humerus became longer than the femur at 29 days and tibiotarsus at 98 days. It became wider than both at approximately 43 days (Figure 3b). Due to the later growth in the wing, the humerus was still growing at 42 days at which point full body mass is reached and the lower limb bones have slowed growing in length (Figure 3a,e). All three bones were still growing radially at this stage based on the growth curves (Figure 3b,f). In the pheasant, the tibiotarsus remained the longest bone and the humerus remained the shortest bone throughout growth (Figure 3c), and maximum growth rates were consistently lower than in the duck. However, the growth period was more prolonged so that after 25 days, longitudinal growth rate for the femur and tibiotarsus was higher in the pheasant than in the duck, and after 30 days radial growth rate for the femur and tibiotarsus was also higher in the pheasant than the duck. The longitudinal growth rates of the femur and humerus were similar (maximum rates 1.00 and 1.10 mm/day) while the tibiotarsus grew slightly faster (maximum rate 1.36 mm/day) (Figure 3g). All three bones reached their maximum longitudinal growth rate at approximately 9 days (femur 9 days, tibiotarsus 10 days, humerus 8 days). In terms of diameter, the humerus grew slightly faster than the other two bones, and reached its maximum radial growth rate (0.0921 mm/day) at 5 days, before the 11‐day estimate for the first flight (Montes et al., 2005), and before the maximum growth rates were reached for the tibiotarsus (0.0638 mm/day) or femur (0.0738 mm/day) at 15 days and 20 days respectively (Figure 3d,h).

### 3.1 Cortical bone growth

**FIGURE 4:**
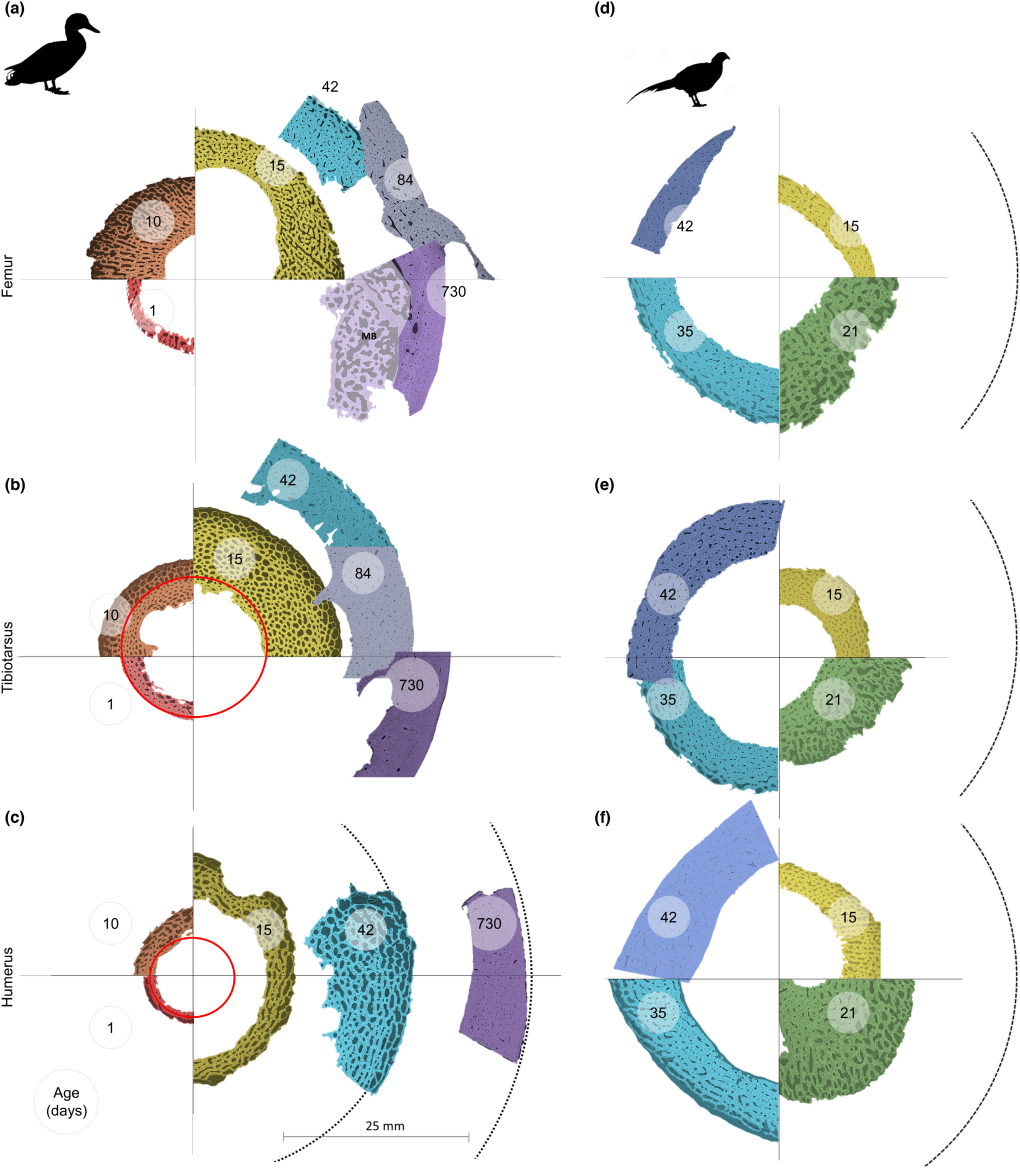
Mid‐diaphyseal transverse virtual sections through the femur, tibiotarsus and humerus at different ages in the domestic duck (*Anas platyrhynchos*) and ring‐necked pheasant (*Phasianus colchicus*). Images show grey value data of single slices from SR CT datasets, pseudo‐coloured to indicate approximate age of the individuals, shown in days (age) for each image. All images are to the same scale and are placed so that the estimated centres for each bone are at the Centre of the cross. (b and c) Area inside red circles indicate the bone region present in the 1‐day‐old individual, referred to here as ‘pre‐hatching bone’. (a) MB indicates medullary bone removed in analysis.

In the duck, the differences in cortical thickness caused by differing rates of endosteal resorption during growth mean that the lower limb keeps a longer record of depositional history during juvenile growth compared with the humerus. For example, at day 1, bone was assumed to have developed largely before hatching (white dashes in Figure 4b,c) with a structure that is visibly distinct from bone deposited later (it has narrower canals). Therefore, bone resembling the structures seen at this stage, and at the same position and scale as the 1‐day‐old individual, is here described as ‘pre‐hatching’ bone (Figure 4). ‘Pre‐hatching bone’ was visible at day 10 in both the tibiotarsus and the humerus (Figure 4b,c). In the tibiotarsus, this region was similar in thickness and appearance to the same region at day 1, while in the humerus, the region was slightly thinner than at day 1 and was not present in places, likely due to endosteal resorption. At day 15, no ‘pre‐hatching bone’ was visible in the humerus (Figure 4c), while a thin layer was still present on the endosteal surface of the tibiotarsus. Also worth noting is the presence of medullary bone within the adult femur, which indicates a high degree of bone turnover and likely remodelling related to egg laying (Figure 4a)—this is included in the image for visual purposed but medullary bone tissue was removed from all quantitative analyses.

**FIGURE 5:**
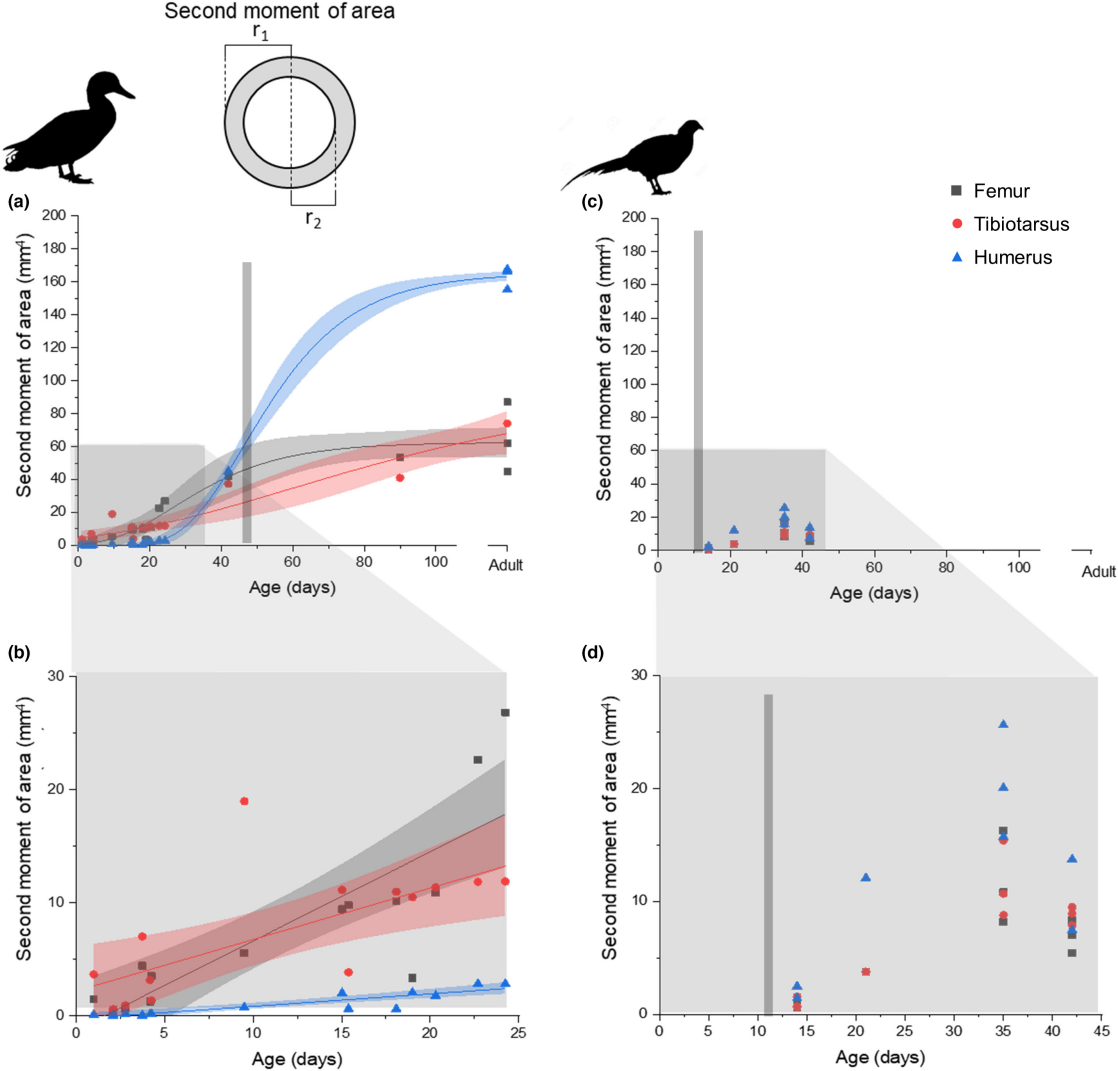
Second moment of area for the femur, tibiotarsus and humerus at different ages across (a and c) the entire age range and (b and d) juvenile growth in domestic ducks (*Anas platyrhyncos*), and ring‐necked pheasant (*Phasianus colchicus*). Vertical grey lines indicate approximate age of flight. (a) Lines indicate Gompertz curve fit and shaded coloured regions indicate 95% confidence intervals for the fit. (b) Lines indicate linear fit and shaded coloured regions indicate 95% confidence intervals for the fit.

Radial bone growth was measured using the diameters already discussed, and the thickness of the bone cortex, which in combination give the second moment of area. At first in the duck, the cortical thickness increased more rapidly in the tibiotarsus and femur (Figure 4c), overshooting the cortical thickness present in adults for both bones. Combined with the earlier radial growth, this resulted in a greater second moment of area in the lower limb bones compared with the humerus, which at 24 days had a second moment of area 10 times smaller than the femur (Figure 5a). At 42 days, the cortical thickness was reduced in the tibiotarsus and femur and greatly increased in the humerus, along with the diameter, so that at this stage the second moment of area was a little higher in the humerus than the tibiotarsus (Figure 5a). The change in second moment of area between the largest juvenile and sub‐adult was 28% for the tibiotarsus, but 4400% for the humerus. This rapid growth continued as in the adult the second moment of area was twice as high in the humerus compared with the lower limb bones, representing a further growth of 226% in terms of second moment of area while the tibiotarsus increased by 84%. In the pheasant, we do not have adult individuals, but the juveniles growth patterns were different. Second moment of area was highest in the humerus compared to the other two bones from the youngest individuals measured (14 days) and increased in all three bones to 35 days. However, on average the second moment of area was lower for the three bones in the 42‐day‐old individuals than in the 35‐day‐old individuals due to reduced cortical thickness. In the duck, second moment of area was never found to decrease, only increase.

### 3.2 Porosity

**FIGURE 6:**
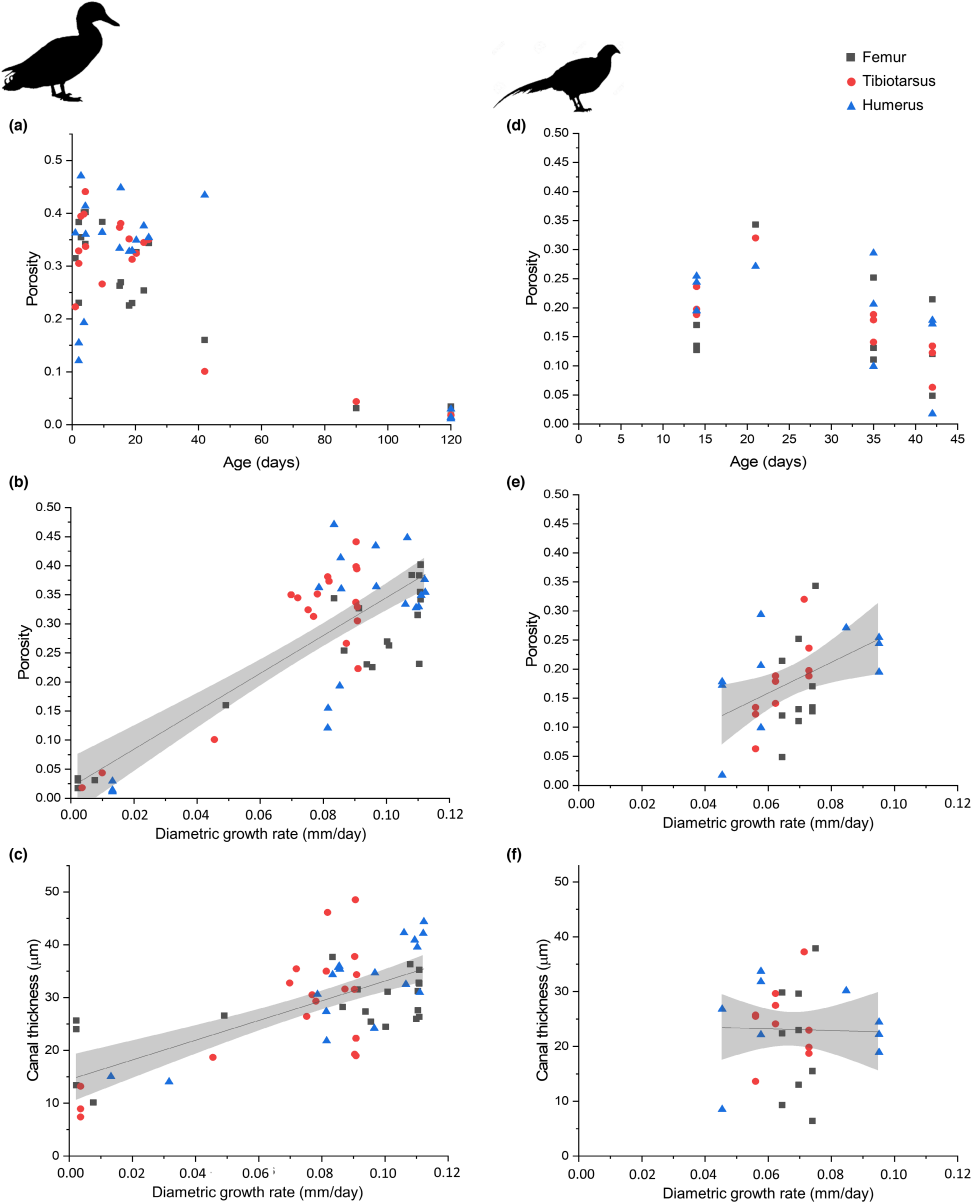
Mid‐diaphyseal cortical porosity and mean canal thickness in the femur, tibiotarsus and humerus (blue triangles) domestic ducks (*Anas platyrhynchos*) and ring‐necked pheasants (*Phasianus colchicus*). (a–f) Femur: Grey squares, tibiotarsus: Red circles and humerus: Blue triangles. (a) Consistent porosity at all juvenile ages in all three bones. (b and e) relationship between growth rate and porosity. (c and f) Relationship between growth rate and canal thickness. Lines show linear regression with 95% confidence intervals for display purposes.

In the juvenile ducks, there was no significant correlation between age and porosity in any of the bones (Figure 6a). In the pheasants, mean porosity was greatest at 21 days, with lower porosity both before and after this age stage (Figure 6b), though again there was no significant correlation between age and porosity. In both ducks and pheasants, there was a positive correlation between diametric growth rate and bone porosity for most bones (Figure 6b,e). There was a positive relationship between diametric growth rate and bone porosity for the duck humerus (*p* < 0.001, pseudo *r* ^2^ = 0.831), tibiotarsus (*p* < 0.001, pseudo *r* ^2^ = 0.866) and femur (*p* < 0.001, pseudo *r* ^2^ = 0.779). For the pheasant, the same relationship was present in the humerus (*p* < 0.05, pseudo *r* ^2^ = 0.290) and tibiotarsus T (*p* < 0.001, pseudo *r* ^2^ = 0.607), but not the femur.

In the ducks, a linear relationship was also present between diametric growth rate and mean canal thickness (Figure 6c) (*p* < 0.0001, *r* ^2^ = 0.482). This growth rate/canal thickness relationship was not observed in the pheasant though fewer age stages were present (Figure 6f).

### 3.3 Canal orientations

**FIGURE 7:**
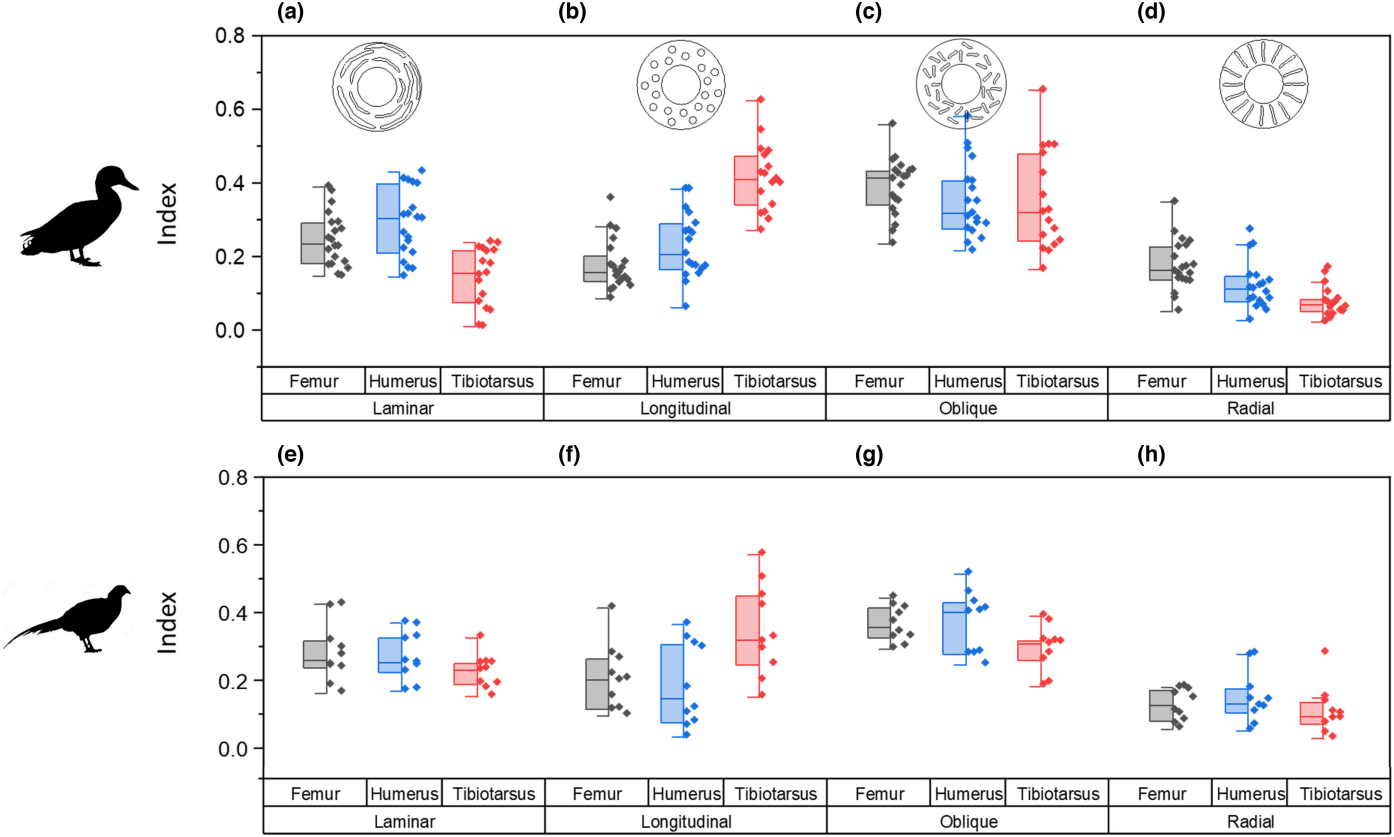
Major orientations of vascular canals across all ages in the mid‐diaphyseal region of the bone cortex in the femur, tibiotarsus and humerus of domestic ducks (*Anas platyrhynchos*) (a‐d) and ring‐necked pheasant (*Phasianus colchicus*) (e–h). (a–h) Proportion of vascular canals is defined as (a and e) laminar, (b and f) longitudinal, (c and g) oblique and (d and h) radial. Box shows interquartile range and whiskers show 1.5 times the interquartile range.

In the duck, pooling all ages, the tibiotarsus had a smaller laminar index (proportion of laminar vascular canals) (Figure 7a) and a larger longitudinal index (proportion of longitudinal vascular canals) (Figure 7b) than the humerus (*p <* 0.001 and *p* < 0.001, respectively) or femur (*p* < 0.001 and *p* < 0.05 respectively). The oblique index was consistent across the three bones (Figure 6c). The femur had a higher radial index than the tibiotarsus (*p* < 0.05), (Figure 7d).

In the pheasant, the patterns and magnitudes of the indices were similar to the duck, though the tibiotarsus did not show a reduced radial index compared to the femur and humerus. The longitudinal index was higher in the tibiotarsus than either the femur (*p* < 0.05) or the humerus (*p* < 0.01).

**FIGURE 8:**
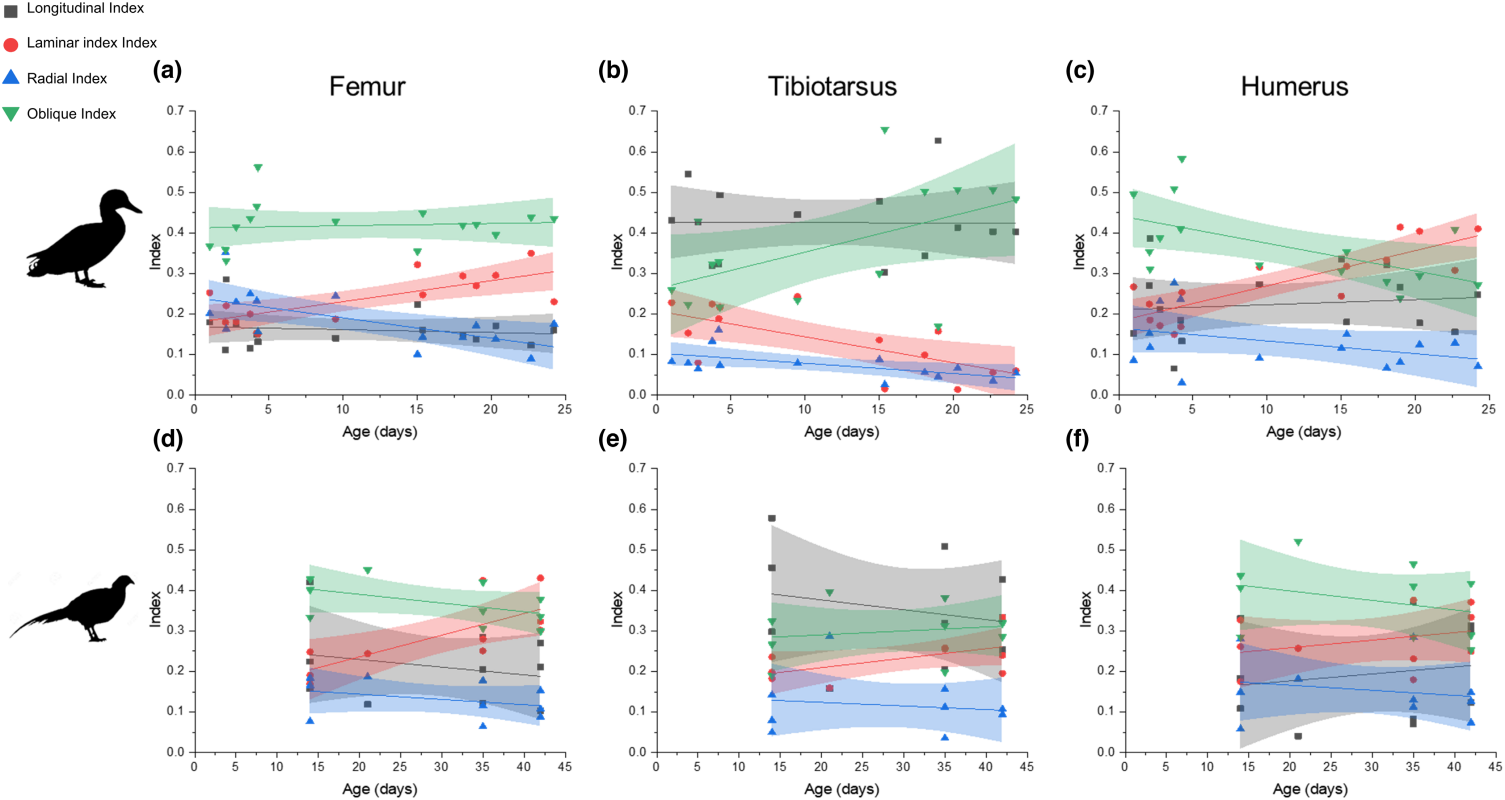
Major vascular canal orientations in the mid‐diaphyseal cortical bone of the femur, tibiotarsus and humerus at different ages in the domestic duck (*Anas platyrhynchos*) (a–c) and ring‐necked pheasant (*Phasianus colchicus*) (d–f). Proportion of canals that are longitudinal, laminar, oblique and radial in the (a and d) femur, (b and e) tibiotarsus and (c and f) humerus at different ages in juvenile birds. Lines show linear regressions and 95% confidence limits for each data set for display purposes.

In the femura and humeri of juvenile ducks, the laminar index increased significantly with age (Figure **8**a,c; pseudo *r* ^2^ = 0.43 and 0.643, *p* < 0.01 and 0.001, respectively), while the oblique index decreased (pseudo *r* ^2^ = 0.21 and 0.372, *p* < 0.05 and 0.01, respectively). No statistically significant correlation was observed between age and either longitudinal index or radial index for the humerus, but in the femur, the longitudinal index increased (pseudo *r* ^2^ = 0.5, *p* < 0.001) (Figure 8a). In the tibiotarsus, the relationship between age and orientation was weaker but still significant. The laminar index and radial index decreased and the oblique index increased with age (pseudo *r* ^2^ = 0.38, 0.39, 0.30 and *p* < 0.001, <0.01, <0.001, respectively) (Figure 8b). In pheasants, the only index that changed significantly with age was the laminar index which increased with age in the femur (*p* < 0.001, pseudo *r* ^2^ = 0.602) and to a lesser degree in the tibiotarsus (*p* < 0.05, pseudo *r* ^2^ = 0.330).

Across all ages in the duck humerus and tibiotarsus, no relationship was found between growth rate and canal orientation, in the femur increased growth rates were positively associated with radial index (*p* < 0.001, pseudo *r* ^2^ = 0.789) and negatively associated with laminar index (*p* < 0.01, pseudo *r* ^2^ = 0.229). In pheasants, the laminar index in the femur decreased with growth rate (*p* < 0.01, pseudo *r* ^*2*^ = 0.504), while the oblique index increased (*p* < 0.05, pseudo *r* ^2^ = 0.318). In the pheasant tibiotarsus, the laminar index decreased with growth rate (*p* < 0.05, pseudo *r* ^2^ = 0.347) but there was no correlation with oblique index and no change in laminar index was found with growth rate in the humerus.

## 4 DISCUSSION

Both ducks and pheasants are precocial, but their developmental patterns are different, as previously described by Montes et al. (2005). The growth curves produced here closely matched those described by Montes et al. (2005), showing that although the curves for the pheasant were less complete than those for ducks, the fits produced are likely to be accurate (Figure 3). The ducks had faster growth rates and reached full size in approximately 7 weeks (Figure 3a,e), while the pheasants had slower but more protracted growth periods (Figure 3c,g). Wild bird species show a wide variation in growth rates, but as these birds are domestic, part of the difference in growth rate may be due to selective breeding for higher body masses—wild mallards have an adult body mass of approximately 1 kg while domestic ducks have an approximate body mass of 2 kg (Giammarino & Quatto, 2017). Therefore the exact results we see may not be representative of wild birds. Their growth patterns, however, are very similar with rapid growth leading to acquisition of full body mass in approximately 55–60 days (Giammarino & Quatto, 2017). Even in wild mallards, most growth occurs earlier than in pheasants, indicating a potential adaptive difference in growth patterns. As pheasants can fly to escape predators earlier in development than ducks, it may be that rapid growth in ducks reduces vulnerability to predators.

The differences in growth patterns and behaviour also result in variation between the bones of the wing and leg. In the duck, the growth rate of the lower limb bones was rapid initially but decreased within the first week of growth, while the humerus reached it maximum growth rate much later, at 24 days (Figure 3e). In the pheasant, the growth patterns for the three bones were more consistent, with all three reaching their maximum longitudinal growth rate in approximately 9 days (Figure 3g). This variation matches with the development of walking and flight behaviours in the two species: both species begin to walk from the day of hatching, so the tibiotarsus and femur are used for walking immediately. However, ducklings cannot fly until 56 days of age (Montes et al., 2005) so the humerus is not used for flight until this point (Prondvai et al., 2018), and it has been suggested that the upper limb follows a more altricial developmental pattern compared with the legs (Dial & Carrier, 2012; Prondvai et al., 2018). In pheasants, flight also does not occur from hatching but develops much earlier than in ducks, from around 11 days (Montes et al., 2005), so wing bones are likely to be in use from this stage. In ducks, the longest adult bone is the humerus, while in pheasants, the humerus is shorter than the tibiotarsus, likely reflecting the differences in flight capabilities between the two species: ducks are strong fliers while pheasants fly in short bursts.

In the femur and tibiotarsus of the duck, cortical thickness and second moment of area (which is related to resistance to bending) increased with age from early in growth (Figure 5) (and body mass), while canal thickness also increased (Figure 6), suggesting rapid bone deposition to create a thick cortex while retaining a porous structure until later in growth. This has previously been observed in the California gull, where hindlimb cortical thickness compensated for relatively weak bone tissue to maintain a constant breaking force for the bone, relative to body mass (Carrier & Leon, 1990).

In the duck humerus, no correlations were found between age and vascular dimensions or porosity (Figure 6). Like in the California gull (Carrier & Leon, 1990), mechanical requirements are less important in the wing than the leg in early growth since juvenile ducks are flightless. Therefore, the rapidly growing bone does not need the same strength that is required by the lower limb bones (Prondvai et al., 2018), as further demonstrated by the slower increase in second moment of area (Figure 5). The bone can grow rapidly without the requirement for particular strength, and then becomes stronger (three to four times stronger in the California gull (Carrier & Leon, 1990) and more than 40 times higher second moment of area here) only when the limb is required for flight, here by approximately 42 days when the second moment of area becomes greater in the humerus than the femur tibiotarsus for the first time (Figure 5).

In the pheasant, growth was slower but more protracted (Figure 3c,d,g,h) and in general bone tissue is less porous (Figure 6). Unlike in the duck which shows constant increase in second moment of area for all the bones, the samples tested here suggest an increase in second moment of area followed by a decrease (Figure 5). However, this pattern is also mirrored by changes in bone porosity—as the second moment of area decreases so does the porosity (Figure 6). Second moment of area was here calculated using the radius of the midshaft of the bone and the cortical thickness, it did not take into account the porosity and is therefore an overestimate of the true second moment of area. The overestimation will be greater where porosity is higher. Since bone strength is related to both shape and mineralisation/porosity, it is likely that this demonstrates a redistribution of bone tissue from a thicker structure made from more porous but cheap to deposit bone material to a thinner but less porous and therefore stronger structure (Ramchand & Seeman, 2018). Therefore, the strength of the bone may not decrease despite the decrease in second moment of area as estimated here.

In the duck humerus and femur, the proportion of laminar canals was on average higher than in the tibiotarsus (Figure 7), and increased with age within the juvenile age stages (Figure 8), which has also been observed in turkeys (Skedros & Hunt, 2004). Since laminar canals are hypothesized to be useful for resisting torsional loads, this may be related to changing mechanical requirements as the animal ages and, for the wing, begins to fly. Flight in birds places a largely torsional load on the long bones of the wings (Biewener & Dial, 1995; de Margerie, 2002; de Margerie et al., 2005), and in ducks flight occurs only after the bird has reached full body size. On this account, the bone may be growing without the requirement to resist torsional loading until later in growth. However, this pattern was not seen in the pheasant humerus, where no significant change occurred in vascular canal orientation (Figure 7). In homing pigeons, laminarity actually decreases with age (McGuire et al., 2020), and clearly the increase in the duck and pheasant femur laminarity is unlikely to be related to the origin of flight, though it may relate to increasing loads as the animal grows. No relationship was found between age and canal orientation in the tibiotarsus, unlike the emu (Kuehn et al., 2019), where canal orientation became more laminar with age. It is possible that the difference in wing laminarity between ducks and pheasants is due to their difference in flight capability, with ducks being stronger fliers and therefore requiring greater adaptations to flight in their bone microstructure. Flight style has previously been linked to histological differences (Frongia et al., 2021), but this does not explain the reduction in laminarity with age in homing pigeons (McGuire et al., 2020). Additionally, the present dataset for pheasants covers a more limited age range than that for ducks so further work would be required to determine whether these differences are simply an artefact of an incomplete dataset.

The few studies that have characterised actual mechanical strains suggest that shear strains (the localised result of torsion) also occur in the tibiotarsus in both the emu (Main & Biewener, 2007) and the chicken (Biewener et al., 1986), questioning the validity of the hypothesis that laminarity is linked to torsion resistance. Also, both the present study and a recent 3D quantitative study found that in birds the humerus does not necessarily contain more laminar canals than the femur (Pratt et al., 2018), which is expected to be more resistant to bending rather than torsion (Farke & Alicea, 2009), although in the emu at least torsion is also important in the femur (Main & Biewener, 2007). Where strains were measured, only a weak relationship was found between laminarity and bone loading direction in the emu (Kuehn et al., 2019). In addition, bats do not appear to have greater laminarity in wing bones despite apparently similar bone loading (Lee & Simons, 2015; Pratt et al., 2018). However, laminarity does appear to correlate with flight styles, with species with flight styles predicted to increase torsional loading having higher cortical laminarity (Frongia et al., 2021; Simons & O'connor, 2012).

In the present study, the pattern is not easy to interpret: the increase in laminarity occurs long before the bird starts to fly and during the period where the second moment of area is low, suggesting limited use of the wing, and also occurs in the femur. Further study is required to assess how and if laminarity could affect bone mechanics and how prevalent the varying orientations are in different birds at different ontogenetic stages. In addition, actual strain measurements would be required for ducks to measure the directions of loading in life.

Canal orientation has also been correlated with bone tissue deposition rate (Castanet et al., 2000; Pratt & Cooper, 2018). Usually, radial canals are associated with increased growth rate, and laminar canals with a decreased growth rate (Castanet et al., 2000, Pratt & Cooper, 2018). However here, canal orientation was not associated with growth rate in either the tibiotarsus or humerus in either species. In the femur, increased growth rate was associated with more oblique canals and fewer longitudinal and laminar canals in the duck, and fewer laminar canals in the pheasant. Given the growth of the humerus is delayed relative to the lower limb bones, it is predicted that there will be a greater proportion of radial canals later in the development of the humerus, as at this stage the bone grows rapidly to catch up with the lower limb bones. This was not found here: in the 24‐day‐old bird, when humerus growth should be fastest, the radial index in the humerus was only 0.0708, while the laminar index was 0.410 (Figure 8). In the tibiotarsus at this age, the laminar and radial indices were 0.0601 and 0.0539, respectively, despite the bone growing more slowly. In addition, the femur had a higher radial index than the tibiotarsus despite growing at a similar appositional rate. The femur was generally more similar to the humerus in terms of canal organisation than to the tibiotarsus despite their very different growth patterns. A previous study investigating the relationship between canal orientation and bone deposition rate in the duck also found no relationship (de Margerie et al., 2002), contrary to studies in other species (de Margerie et al., 2004; Pratt & Cooper, 2018), so it is possible that duck bones differ from those of other birds. Again, more work is needed to study the relationships between microstructure, deposition rate and bone mechanics, and in particular how those bone characteristics interact.

The observations from this study provide further information that could help in the interpretation of fossil bone. For example, in all bones studied, porosity decreased from juvenile, to sub‐adult and again to adult age stages, demonstrating the bone deposition and infilling process. A similar process has also been observed in mice (Bortel et al., 2015) and humans (Ramchand & Seeman, 2018). In the duck, it is possible to use the porosity values to distinguish between adult, sub‐adult and juvenile, based on the tibiotarsus, but since porosity was related to growth rate, the result from the humerus may differ, as also found across fossils (Prondvai et al., 2018). Since growth rates vary between species and hence, porosity also varies between species, absolute values of porosity or canal diameter will not be directly comparable for estimating maturity across species. However, it may be possible to develop a metric that takes into account both porosity and spacing/separation of canals which could give an indication of the initial bone tissue deposition pattern. It would then be possible to infer maturity from a combination of the initial bone tissue deposition pattern and canal thickness, which would indicate how much infilling had occurred. Additionally, the presence of distinctly different pre‐hatching bone could be used to distinguish very early growth from later growth, though this would only work in bones where less endosteal resorption occurs (i.e., tibiotarsus rather than humerus). However, intraskeletal variation should be investigated further, both between different bones and between different regions of the same bone, since vascular organisation has been shown to vary between different regions of the same bone in mice (Núñez et al., 2018), and canal orientations may vary in different regions (de Margerie et al., 2004).

It is worth noting that this study was carried out in domestic animals, which have been demonstrated to have differences in bone structure compared to wild animals due to differences in exercise, diet and stresses (Harbers et al., 2020; Zack et al., 2021), but given that all the animals in this study were domestic, this is at least consistent within the study. Perhaps a larger issue with the opportunistic sampling approach taken is that there may be underlying health problems in the birds studied which may cause differences in their bone structure compared with healthy individuals—this should be studied further in the future—however, we chose to take the most ethical approach we could for this study by using animals that were naturally deceased.

## 5 CONCLUSIONS

This study characterises 3D bone microstructure through development in two extant avian species, the domestic duck and ring‐necked pheasant. The results demonstrate differing growth patterns in the leg and wing in the duck but not the pheasant. These differences have been described before but not using a histological approach through development, and not with the additional insight that a 3D approach con provide especially with regards to improved quantification of vascular canal orientation. These results are important for understanding bone growth in birds, but also highlight some important caveats for the interpretation of fossils: data on, for example, age obtained from the leg bones may differ significantly from those obtained from the wing. If this mosaic of characteristics is also present in fossil material, it is critically important to study several different regions from the same fossil. This approach could also provide additional information about, for example, timings of walking and flying behaviours. Measures of vascular canal orientation suggested that the mean canal orientation changed through development in the duck, which may relate to changing mechanical function of the bone though further work is required to test this hypothesis and in species other than the duck, and in a full growth series in pheasants, and possibly comparing the domestic ducks seen here with wild mallards. This study therefore provides new insights into avian bone growth and comparative data that will aid in the interpretation of fossil material.

## AUTHOR CONTRIBUTIONS

KAW, ROCO, NJG and PS designed the study, KAW carried out the experiments and analyses, and KAW and PS wrote and revised the paper. All authors had editorial input on the paper and contributed to discussions throughout the study.

## Supporting information


Figure S1

## Data Availability

All data supporting this study will be openly available from the University of Southampton institutional research repository on publication at https://doi.org/10.5258/SOTON/D1805.

## References

[joa13741-bib-0001] Biewener, A. & Dial, K. (1995) In vivo strain in the humerus of pigeons (Columba livia) during flight. Journal of Morphology, 225, 61–75.

[joa13741-bib-0002] Biewener, A.A. , Swartz, S.M. & Bertram, J.E. (1986) Bone modeling during growth: dynamic strain equilibrium in the chick tibiotarsus. Calcified Tissue International, 39, 390–395. 3100003 10.1007/BF02555177

[joa13741-bib-0003] Bortel, E.L. , Duda, G.N. , Mundlos, S. , Willie, B.M. , Fratzl, P. & Zaslansky, P. (2015) Long bone maturation is driven by pore closing: A quantitative tomography investigation of structural formation in young C57BL/6 mice. Acta Biomaterialia, 22, 92–102. 25829108 10.1016/j.actbio.2015.03.027

[joa13741-bib-0004] Carrier, D. & Leon, L.R. (1990) Skeletal growth and function in the California gull (Larus californicus). Journal of Zoology, 222, 375–389.

[joa13741-bib-0005] Castanet, J. , Rogers, K.C. , Cubo, J. & Boisard, J.J. (2000) Periosteal bone growth rates in extant ratites (ostriche and emu). Implications for assessing growth in dinosaurs. Comptes Rendus De L Academie Des Sciences Serie Iii‐Sciences De La Vie‐Life Sciences, 323, 543–550. 10.1016/s0764-4469(00)00181-5 10923210

[joa13741-bib-0006] Cohen, A. & Serjeantson, D. (1996) A manual for the identification of bird bones from archaeological sites. London, UK: Archetype Publications.

[joa13741-bib-0007] Cooper, D.M. , Kawalilak, C.E. , Harrison, K. , Johnston, B.D. & Johnston, J.D. (2016) Cortical bone porosity: what is it, why is it important, and how can we detect it? Current Osteoporosis Reports, 14, 187–198. 27623679 10.1007/s11914-016-0319-y

[joa13741-bib-0008] De Boef, M. & Larsson, H.C.E. (2007) Bone microstructure: quantifying bone vascular orientation. Canadian Journal of Zoology‐Revue Canadienne De Zoologie, 85, 63–70.

[joa13741-bib-0009] De Margerie, E. (2002) Laminar bone as an adaptation to torsional loads in flapping flight. Journal of Anatomy, 201, 521–526. 12489764 10.1046/j.1469-7580.2002.00118.x PMC1570990

[joa13741-bib-0010] De Margerie, E. , Cubo, J. & Castanet, J. (2002) Bone typology and growth rate: testing and quantifying 'Amprino's rule' in the mallard (*Anas platyrhynchos*). Comptes Rendus Biologies, 325, 221–230. 12017770 10.1016/s1631-0691(02)01429-4

[joa13741-bib-0011] De Margerie, E. , Robin, J.P. , Verrier, D. , Cubo, J. , Groscolas, R. & Castanet, J. (2004) Assessing a relationship between bone microstructure and growth rate: a fluorescent labelling study in the king penguin chick (*Aptenodytes patagonicus*). Journal of Experimental Biology, 207, 869–879. 14747417 10.1242/jeb.00841

[joa13741-bib-0012] De Margerie, E. , Sanchez, S. , Cubo, J. & Castanet, J. (2005) Torsional resistance as a principal component of the structural design of long bones: comparative multivariate evidence in birds. The Anatomical Record, 282, 49–66. 15584036 10.1002/ar.a.20141

[joa13741-bib-0013] Dial, T.R. & Carrier, D.R. (2012) Precocial hindlimbs and altricial forelimbs: partitioning ontogenetic strategies in mallards (*Anas platyrhynchos*). Journal of Experimental Biology, 215, 3703–3710. 22855613 10.1242/jeb.057380

[joa13741-bib-0014] Doube, M. , Kłosowski, M.M. , Arganda‐Carreras, I. , Cordelières, F.P. , Dougherty, R.P. , Jackson, J.S. et al. (2010) BoneJ: free and extensible bone image analysis in ImageJ. Bone, 47, 1076–1079. 20817052 10.1016/j.bone.2010.08.023 PMC3193171

[joa13741-bib-0015] Farke, A.A. & Alicea, J. (2009) Femoral strength and posture in terrestrial birds and non‐avian theropods. The Anatomical Record: Advances in Integrative Anatomy and Evolutionary Biology: Advances in Integrative Anatomy and Evolutionary Biology, 292, 1406–1411. 10.1002/ar.20963 19711474

[joa13741-bib-0016] Frongia, G.N. , Naitana, S. , Farina, V. , Gadau, S.D. , Stefano, M.D. , Muzzeddu, M. et al. (2021) Correlation between wing bone microstructure and different flight styles: the case of the griffon vulture (*Gyps fulvus*) and greater flamingo (*Phoenicopterus roseus*). Journal of Anatomy, 239(1), 59–69. 33650143 10.1111/joa.13411 PMC8197951

[joa13741-bib-0017] Gao, C.L. , Chiappe, L.M. , Zhang, F.J. , Pomeroy, D.L. , Shen, C.Z. , Chinsamy, A. et al. (2012) A subadult specimen of the early cretaceous bird Sapeornis chaoyangensis and a taxonomic reassessment of Sapeornithids. Journal of Vertebrate Paleontology, 32, 1103–1112.

[joa13741-bib-0018] Giammarino, M. & Quatto, P. (2017) Growth curves of wild mallard, based on functional analysis of capture–recapture data. Ringing & Migration, 32, 37–42.

[joa13741-bib-0019] Griffin, C.T. & Nesbitt, S.J. (2020) Does the maximum body size of theropods increase across the Triassic–Jurassic boundary? Integrating ontogeny, phylogeny, and body size. The Anatomical Record, 303, 1158–1169. 30968581 10.1002/ar.24130

[joa13741-bib-0020] Harbers, H. , Zanolli, C. , Cazenave, M. , Theil, J.‐C. , Ortiz, K. , Blanc, B. et al. (2020) Investigating the impact of captivity and domestication on limb bone cortical morphology: an experimental approach using a wild boar model. Scientific Reports, 10, 1–13. 33149160 10.1038/s41598-020-75496-6 PMC7643176

[joa13741-bib-0021] Kuehn, A.L. , Lee, A.H. , Main, R.P. & Simons, E.L. (2019) The effects of growth rate and biomechanical loading on bone laminarity within the emu skeleton. PeerJ, 7, e7616. 31579580 10.7717/peerj.7616 PMC6765378

[joa13741-bib-0022] Lee, A.H. & Simons, E.L.R. (2015) Wing bone laminarity is not an adaptation for torsional resistance in bats. Integrative and Comparative Biology, 55, E108. 10.7717/peerj.823 PMC4359045 25780775

[joa13741-bib-0023] Li, C.H. & Tam, P.K.S. (1998) An iterative algorithm for minimum cross entropy thresholding. Pattern Recognition Letters, 19(8), 771–776.

[joa13741-bib-0024] Main, R.P. & Biewener, A.A. (2007) Skeletal strain patterns and growth in the emu hindlimb during ontogeny. Journal of Experimental Biology, 210, 2676–2690. 17644682 10.1242/jeb.004580

[joa13741-bib-0025] Maiorino, L. , Farke, A.A. , Kotsakis, T. & Piras, P. (2013) Is Torosaurus triceratops? Geometric morphometric evidence of late Maastrichtian Ceratopsid dinosaurs. Plos One, 8(11), e81608. 24303058 10.1371/journal.pone.0081608 PMC3841114

[joa13741-bib-0026] Marone, F. & Stampanoni, M. (2012) Regridding reconstruction algorithm for real‐time tomographic imaging. Journal of Synchrotron Radiation, 19, 1029–1037. 23093766 10.1107/S0909049512032864 PMC3480277

[joa13741-bib-0027] Marotti, G. (2010) Static and dynamic osteogenesis. Italian Journal of Anatomy and Embryology, 115, 123–126. 21073001

[joa13741-bib-0028] Mcguire, R.S. , Ourfalian, R. , Ezell, K. & Lee, A.H. (2020) Development of limb bone laminarity in the homing pigeon (*Columba livia*). PeerJ, 8, e9878. 33194361 10.7717/peerj.9878 PMC7485507

[joa13741-bib-0029] Montes, L. , DE Margerie, E. , Castanet, J. , DE Ricqlès, A. & Cubo, J. (2005) Relationship between bone growth rate and the thickness of calcified cartilage in the long bones of the Galloanserae (Aves). Journal of Anatomy, 206, 445–452. 15857365 10.1111/j.1469-7580.2005.00410.x PMC1571507

[joa13741-bib-0030] Norberg, R.Å. & Aldrin, B.S.W. (2010) Scaling for stress similarity and distorted‐shape similarity in bending and torsion under maximal muscle forces concurs with geometric similarity among different‐sized animals. Journal of Experimental Biology, 213, 2873–2888. 20675557 10.1242/jeb.044180

[joa13741-bib-0031] Núñez, J. , Goring, A. , Javaheri, B. , Razi, H. , Gomez‐Nicola, D. , Hesse, E. et al. (2018) Regional diversity in the murine cortical vascular network is revealed by synchrotron X‐ray tomography and is amplified with age. European Cells and Materials, 35, 281–299. 29790567 10.22203/eCM.v035a20

[joa13741-bib-0032] Palumbo, C. , Ferretti, M. , De Pol, A. & Marotti, G. (2002) Apoptosis during static and dynamic bone formation. Journal of Bone and Mineral Research, 17, 954.

[joa13741-bib-0033] Pratt, I.V. & Cooper, D.M.L. (2017) A method for measuring the three‐dimensional orientation of cortical canals with implications for comparative analysis of bone microstructure in vertebrates. Micron, 92, 32–38. 27855318 10.1016/j.micron.2016.10.006

[joa13741-bib-0034] Pratt, I.V. & Cooper, D.M.L. (2018) The effect of growth rate on the three‐dimensional orientation of vascular canals in the cortical bone of broiler chickens. Journal of Anatomy, 233, 531–541. 30022496 10.1111/joa.12847 PMC6131975

[joa13741-bib-0035] Pratt, I.V. , Johnston, J.D. , Walker, E. & Cooper, D.M.L. (2018) Interpreting the three‐dimensional orientation of vascular canals and cross‐sectional geometry of cortical bone in birds and bats. Journal of Anatomy, 232, 931–942. 29520776 10.1111/joa.12803 PMC5979616

[joa13741-bib-0036] Prondvai, E. , Godefroit, P. , Adriaens, D. & Hu, D.‐Y. (2018) Intraskeletal histovariability, allometric growth patterns, and their functional implications in bird‐like dinosaurs. Scientific Reports, 8, 1–16. 29321475 10.1038/s41598-017-18218-9 PMC5762864

[joa13741-bib-0037] Ramchand, S.K. & Seeman, E. (2018) The influence of cortical porosity on the strength of bone during growth and advancing age. Current Osteoporosis Reports, 16, 561–572. 30187285 10.1007/s11914-018-0478-0

[joa13741-bib-0038] Rauhut, O.W. , Foth, C. & Tischlinger, H. (2018) The oldest archaeopteryx (Theropoda: Avialiae): a new specimen from the Kimmeridgian/Tithonian boundary of Schamhaupten, Bavaria. PeerJ, 6, e4191. 29383285 10.7717/peerj.4191 PMC5788062

[joa13741-bib-0039] Rueden, C.T. , Schindelin, J. , Hiner, M.C. , Dezonia, B.E. , Walter, A.E. , Arena, E.T. et al. (2017) ImageJ2: ImageJ for the next generation of scientific image data. BMC Bioinformatics, 18, 529. 29187165 10.1186/s12859-017-1934-z PMC5708080

[joa13741-bib-0040] Scannella, J.B. & Horner, J.R. (2010) Torosaurus marsh, 1891, is triceratops marsh, 1889 (Ceratopsidae: Chasmosaurinae): synonymy through ontogeny. Journal of Vertebrate Paleontology, 30, 1157–1168.

[joa13741-bib-0041] Simons, E.L. & O'connor, P.M. (2012) Bone laminarity in the avian forelimb skeleton and its relationship to flight mode: testing functional interpretations. The Anatomical Record: Advances in Integrative Anatomy and Evolutionary Biology, 295, 386–396. 22241723 10.1002/ar.22402

[joa13741-bib-0042] Skedros, J.G. & Hunt, K.J. (2004) Does the degree of laminarity correlate with site‐specific differences in collagen fibre orientation in primary bone? An evaluation in the Turkey ulna diaphysis. Journal of Anatomy, 205, 121–134. 15291795 10.1111/j.0021-8782.2004.00318.x PMC1571335

[joa13741-bib-0043] Starck, J.M. & Chinsamy, A. (2002) Bone microstructure and developmental plasticity in birds and other dinosaurs. Journal of Morphology, 254, 232–246. 12386894 10.1002/jmor.10029

[joa13741-bib-0044] Stein, K.W. & Werner, J. (2013) Preliminary analysis of osteocyte lacunar density in long bones of tetrapods: all measures are bigger in sauropod dinosaurs. PLoS One, 8, e77109. 24204748 10.1371/journal.pone.0077109 PMC3812986

[joa13741-bib-0045] Vincenzi, S. , Jesensek, D. & Crivelli, A.J. (2020) Biological and statistical interpretation of size‐at‐age, mixed‐effects models of growth. Royal Society Open Science, 7, 192146. 32431890 10.1098/rsos.192146 PMC7211857

[joa13741-bib-0046] Watson, J.E. & Ledogar, S.H. (2019) Testing the effectiveness of osteometrics in the identification of north American gallinaceous bird post‐cranial elements. Archaeological and Anthropological Sciences, 11, 2623–2636.

[joa13741-bib-0047] Williams, K.A. , Gostling, N.J. , Steer, J. , Oreffo, R. & Schneider, P. (2020) Quantifying intracortical bone microstructure: a critical appraisal of 2D and 3D approaches for assessing vascular canals and osteocyte lacunae. Journal of Anatomy, 238(3), 653–668. 10.1111/joa.13325 PMC7855084 33090473

[joa13741-bib-0048] Winsor, C.P. (1932) The Gompertz curve as a growth curve. Proceedings of the National Academy of Sciences of the United States of America, 18, 1. 16577417 10.1073/pnas.18.1.1 PMC1076153

[joa13741-bib-0049] Zack, E.H. , Smith, S.M. & Angielczyk, K.D. (2021) Effect of captivity on the vertebral bone microstructure of xenarthran mammals. The Anatomical Record, 305(7)1611–1628. 34677912 10.1002/ar.24817

