# Supplementary material for: Ontogenetic changes in cortical bone vascular microstructure in the domestic duck (Anas platyrhynchos) and ring‐necked pheasant (Phasianus colchicus)
Source: J Anat. 2022 Aug 24;241(6):1371–86. doi: 10.1111/joa.13741 (PMC9644950; doi:10.1111/joa.13741)
Supplement: Supplementary file 1 — Figure S1 [file JOA-241-1371-s001.pdf]

## Supplementary material

### Supplementary Methods

Scans were obtained at one of three beamtimes, two at SLS Tomcat, the other at DLS I13-2. For the first SLS Tomcat session, four samples were scanned as a trial at high resolution (0.65 µm voxel size), but this was decreased for later experiments to give a larger field of view (See table S1). During analyses, the pixels were converted to µm so that absolute measurements are comparable.

For DLS datasets, and 0.65 voxel size SLS datasets, scans were slightly noisier than 1.6 µm voxel size SLS datasets, so a Gaussian blur (1 pixel kernel size) was applied before thresholding. Ideally, the same image processing would be applied to both sets of scans, but given the differences in scan quality, it was not possible to find a workflow which was optimised for both.

Images were binarised using a minimum cross entropy thresholding algorithm (Li and Tam, 1998). The bone volume was separated from small image artefacts and noise by a single-voxel erosion and dilation, followed by a component labelling process to remove all except the largest object (*i.e.*, the cortical bone tissue), resulting in a cleaned dataset. The pores within the bone cortex were filled by dilation and erosion operations to create a solid cortical mask. This mask was used to define the edges of the canal network and to extract the intracortical pores (intracortical canals and osteocyte lacunae) by applying an AND function with the inverted cleaned dataset or in other words, by applying the mask to the inverted cleaned dataset that represents the intracortical pores, the medullary cavity and the space around the respective long bone. Intracortical microstructures with volumes of 50-500 µm$^3$ were

considered to be osteocyte lacunae (D'Emic and Benson, 2013) and excluded, while features with volumes >1000 μm$^3$ were classified as vascular canals.

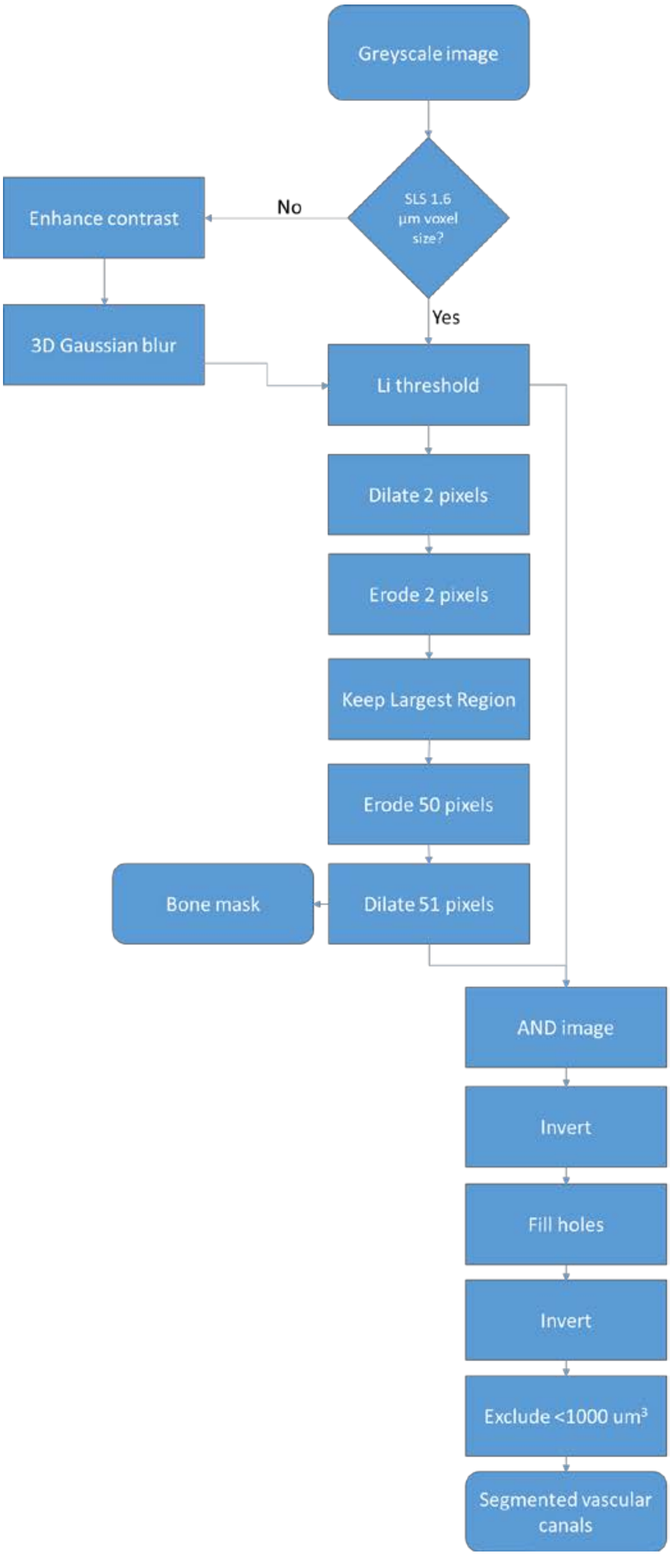


Figure S1: Image processing workflow for segmentation of intracortical vascular canals.

Table S1: Summary of duck tibiotarsus samples imaged during two synchrotron beamtimes.

| **Duck ID** | **Mass (g)** | **Age (days)** | **Bone** | **Beamline** | **Voxel size (µm)** | **Binning** | **Final voxel size (µm)** |
|---|---|---|---|---|---|---|---|
| 2 | 39 | 1 | Tibiotarsus | SLS Tomcat | 0.65 | 2x | 1.3 |
| 4 | 66 | 2.07 | Tibiotarsus | SLS Tomcat | 0.65 | 2x | 1.3 |
| 7 | 67 | 2.11 | Tibiotarsus | SLS Tomcat | 1.6 | n/a | 1.6 |
| 5 | 84 | 2.79 | Tibiotarsus | SLS Tomcat | 1.6 | n/a | 1.6 |
| 6 | 108 | 3.74 | Tibiotarsus | SLS Tomcat | 1.6 | n/a | 1.6 |
| 9 | 119 | 4.18 | Tibiotarsus | SLS Tomcat | 1.6 | n/a | 1.6 |
| 3 | 121 | 4.26 | Tibiotarsus | SLS Tomcat | 0.65 | 2x | 1.3 |
| 1 | 253 | 9.50 | Tibiotarsus | SLS Tomcat | 0.65 | 2x | 1.3 |
| 21 | 392 | 15.02 | Tibiotarsus | SLS Tomcat | 1.6 | n/a | 1.6 |
| 14 | 401 | 15.38 | Tibiotarsus | SLS Tomcat | 1.6 | n/a | 1.6 |
| 10 | 469 | 18.08 | Tibiotarsus | SLS Tomcat | 1.6 | n/a | 1.6 |
| 11 | 492 | 18.00 | Tibiotarsus | SLS Tomcat | 1.6 | n/a | 1.6 |
| 12 | 624 | 24.24 | Tibiotarsus | SLS Tomcat | 1.6 | n/a | 1.6 |
| 20 | 525 | 20.31 | Tibiotarsus | SLS Tomcat | 1.6 | n/a | 1.6 |
| 22 | 585 | 22.69 | Tibiotarsus | SLS Tomcat | 1.6 | n/a | 1.6 |
| 23 | 530 | 20.51 | Tibiotarsus | SLS Tomcat | 1.6 | n/a | 1.6 |
| 13 | 1071 | 42 | Tibiotarsus | SLS Tomcat | 1.6 | n/a | 1.6 |
| 18 | 1900 | 90 | Tibiotarsus | SLS Tomcat | 1.6 | n/a | 1.6 |
| 16 | 1180 | 120 | Tibiotarsus | SLS Tomcat | 1.6 | n/a | 1.6 |

Table S2: Summary of duck humerus samples imaged during two synchrotron beamtimes.

| **Duck ID** | **Mass** | **Age** | **Bone** | **Beamline** | **Voxel size (µm)** | **Binning** | **Final voxel size (µm)** |
|---|---|---|---|---|---|---|---|
| 2 | 39 | 1 | Humerus | SLS | 1.6 | n/a | 1.6 |
| 4 | 66 | 2.07 | Humerus | SLS | 1.6 | n/a | 1.6 |
| 7 | 67 | 2.11 | Humerus | SLS | 1.6 | n/a | 1.6 |
| 5 | 108 | 2.79 | Humerus | SLS | 1.6 | n/a | 1.6 |
| 6 | 84 | 3.74 | Humerus | SLS | 1.6 | n/a | 1.6 |
| 9 | 119 | 4.18 | Humerus | SLS | 1.6 | n/a | 1.6 |
| 3 | 121 | 4.26 | Humerus | SLS | 1.6 | n/a | 1.6 |
| 1 | 253 | 9.50 | Humerus | SLS | 1.6 | n/a | 1.6 |
| 21 | 392 | 15.02 | Humerus | SLS | 1.6 | n/a | 1.6 |
| 14 | 401 | 15.38 | Humerus | SLS | 1.6 | n/a | 1.6 |
| 10 | 469 | 18.08 | Humerus | SLS | 1.6 | n/a | 1.6 |
| 11 | 492 | 18.00 | Humerus | SLS | 1.6 | n/a | 1.6 |
| 12 | 624 | 24.24 | Humerus | SLS | 1.6 | n/a | 1.6 |
| 20 | 525 | 20.31 | Humerus | SLS | 1.6 | n/a | 1.6 |

| | | | | | | | |
|---|---|---|---|---|---|---|---|
| 22 | 585 | 22.69 | Humerus | SLS | 1.6 | n/a | 1.6 |
| 23 | 530 | 20.51 | Humerus | SLS | 1.6 | n/a | 1.6 |
| 13 | 1071 | 42 | Humerus | DLS | 0.8 | 2x | 1.6 |
| 15 | 1181 | 120 | Humerus | SLS | 1.6 | n/a | 1.6 |
| 16 | 1180 | 120 | Humerus | SLS | 1.6 | n/a | 1.6 |
| 19 | 1695 | 120 | Humerus | SLS | 1.6 | n/a | 1.6 |

Table S3: Summary of duck femur samples imaged during two synchrotron beamtimes.

| **Duck ID** | **Mass** | **Age** | **Bone** | **Beamline** | **Voxel size (µm)** | **Binning** | **Final voxel size (µm)** |
|---|---|---|---|---|---|---|---|
| 2 | 39 | 1 | Femur | SLS | 1.6 | n/a | 1.6 |
| 4 | 66 | 2.07 | Femur | SLS | 1.6 | n/a | 1.6 |
| 7 | 67 | 2.11 | Femur | SLS | 1.6 | n/a | 1.6 |
| 5 | 108 | 2.79 | Femur | SLS | 1.6 | n/a | 1.6 |
| 6 | 84 | 3.74 | Femur | SLS | 1.6 | n/a | 1.6 |
| 9 | 119 | 4.18 | Femur | SLS | 1.6 | n/a | 1.6 |
| 3 | 121 | 4.26 | Femur | SLS | 1.6 | n/a | 1.6 |
| 1 | 253 | 9.50 | Femur | SLS | 1.6 | n/a | 1.6 |
| 21 | 392 | 15.02 | Femur | SLS | 1.6 | n/a | 1.6 |
| 14 | 401 | 15.38 | Femur | SLS | 1.6 | n/a | 1.6 |
| 10 | 469 | 18.08 | Femur | SLS | 1.6 | n/a | 1.6 |
| 11 | 492 | 18.00 | Femur | SLS | 1.6 | n/a | 1.6 |
| 12 | 624 | 24.24 | Femur | SLS | 1.6 | n/a | 1.6 |
| 20 | 525 | 20.31 | Femur | SLS | 1.6 | n/a | 1.6 |
| 22 | 585 | 22.69 | Femur | SLS | 1.6 | n/a | 1.6 |
| 23 | 530 | 20.51 | Femur | SLS | 1.6 | n/a | 1.6 |
| 13 | 1071 | 42 | Femur | SLS | 1.6 | n/a | 1.6 |
| 15 | 1181 | 120 | Femur | SLS | 1.6 | n/a | 1.6 |
| 16 | 1180 | 120 | Femur | SLS | 1.6 | n/a | 1.6 |
| 19 | 1695 | 120 | Femur | SLS | 1.6 | n/a | 1.6 |

Table S4: Summary of pheasant femur samples imaged at Diamond Light Source.

| **Pheasant ID** | **Mass (g)** | **Age** | **Bone** | **Beamline** | **Voxel size (µm)** | **Binning** | **Final voxel size (µm)** |
|---|---|---|---|---|---|---|---|
| 2a | 67.5 | 14 | Femur | DLS | 0.8 | 2x | 1.6 |
| 2b | 67 | 14 | Femur | DLS | 0.8 | 2x | 1.6 |
| 2c | 67.6 | 14 | Femur | DLS | 0.8 | 2x | 1.6 |
| 3a | 90 | 21 | Femur | DLS | 0.8 | 2x | 1.6 |
| 5a | 250 | 35 | Femur | DLS | 0.8 | 2x | 1.6 |
| 5b | 300 | 35 | Femur | DLS | 0.8 | 2x | 1.6 |
| 5c | 269 | 35 | Femur | DLS | 0.8 | 2x | 1.6 |

| | | | | | | | |
|---|---|---|---|---|---|---|---|
| 6a | 204.8 | 42 | Femur | DLS | 0.8 | 2x | 1.6 |
| 6b | 292.6 | 42 | Femur | DLS | 0.8 | 2x | 1.6 |
| 6c | 306.7 | 42 | Femur | DLS | 0.8 | 2x | 1.6 |

Table S5: Summary of pheasant humerus samples imaged at Diamond Light Source.

| **Pheasant ID** | **Mass (g)** | **Age** | **Bone** | **Beamline** | **Voxel size (µm)** | **Binning** | **Final voxel size (µm)** |
|---|---|---|---|---|---|---|---|
| 2a | 67.5 | 1 | Humerus | DLS | 0.8 | 2x | 1.6 |
| 2b | 67 | 2.07 | Humerus | DLS | 0.8 | 2x | 1.6 |
| 2c | 67.6 | 2.11 | Humerus | DLS | 0.8 | 2x | 1.6 |
| 3a | 90 | 2.79 | Humerus | DLS | 0.8 | 2x | 1.6 |
| 5a | 250 | 3.74 | Humerus | DLS | 0.8 | 2x | 1.6 |
| 5b | 300 | 4.18 | Humerus | DLS | 0.8 | 2x | 1.6 |
| 5c | 269 | 4.26 | Humerus | DLS | 0.8 | 2x | 1.6 |
| 6a | 204.8 | 9.50 | Humerus | DLS | 0.8 | 2x | 1.6 |
| 6c | 306.7 | 15.38 | Humerus | DLS | 0.8 | 2x | 1.6 |

Table S6: Summary of pheasant tibiotarsus samples imaged at Diamond Light Source.

| **Pheasant ID** | **Mass (g)** | **Age** | **Bone** | **Beamline** | **Voxel size (µm)** | **Binning** | **Final voxel size (µm)** |
|---|---|---|---|---|---|---|---|
| 2a | 67.5 | 14 | Tibiotarsus | DLS | 0.8 | 2x | 1.6 |
| 2b | 67 | 14 | Tibiotarsus | DLS | 0.8 | 2x | 1.6 |
| 2c | 67.6 | 14 | Tibiotarsus | DLS | 0.8 | 2x | 1.6 |
| 3a | 90 | 21 | Tibiotarsus | DLS | 0.8 | 2x | 1.6 |
| 5a | 250 | 35 | Tibiotarsus | DLS | 0.8 | 2x | 1.6 |
| 5b | 300 | 35 | Tibiotarsus | DLS | 0.8 | 2x | 1.6 |
| 5c | 269 | 35 | Tibiotarsus | DLS | 0.8 | 2x | 1.6 |
| 6a | 204.8 | 42 | Tibiotarsus | DLS | 0.8 | 2x | 1.6 |
| 6b | 292.6 | 42 | Tibiotarsus | DLS | 0.8 | 2x | 1.6 |
| 6c | 306.7 | 42 | Tibiotarsus | DLS | 0.8 | 2x | 1.6 |

| Scan | | Mass | Age | Duck ID | Bone | Flen | Tlen | Hlen |
|---|---|---|---|---|---|---|---|---|
| 20160807_1249_KW_ | | 39 | 1 | 2 | f | 1.765944 | 3.029688 | 1.197792 |
| 20160807_ | SLS | 39 | 1 | 2 | h | 1.765944 | 3.029688 | 1.197792 |
| D2TBoneV | SLS | 39 | 1 | 2 | t | 1.765944 | 3.029688 | 1.197792 |
| 20160807_ | SLS | 66 | 2.072745 | 4 | f | 2.056 | 3.832 | 1.256 |
| 20160807_ | SLS | 66 | 2.072745 | 4 | h | 2.056 | 3.832 | 1.256 |
| D4TBoneV | SLS | 66 | 2.072745 | 4 | t | 2.056 | 3.832 | 1.256 |
| 20160807_ | SLS | 67 | 2.112475 | 7 | t | 2.056 | 3.832 | 1.256 |
| 20160807_ | SLS | 67 | 2.112475 | 7 | f | 2.056 | 3.832 | 1.256 |
| 20160807_ | SLS | 67 | 2.112475 | 7 | h | 2.056 | 3.832 | 1.256 |
| 20160807_ | SLS | 84 | 2.787882 | 5 | f | 2.032 | 3.888 | 1.28 |
| 20160807_ | SLS | 108 | 2.787882 | 5 | h | 2.13516 | 3.607632 | 1.307232 |
| 20160807_ | SLS | 84 | 2.787882 | 5 | t | 2.032 | 3.888 | 1.28 |
| 20160807_ | SLS | 108 | 3.741398 | 6 | t | 2.13516 | 3.607632 | 1.307232 |
| 20160807_ | SLS | 108 | 3.741398 | 6 | f | 2.13516 | 3.607632 | 1.307232 |
| 20160807_ | SLS | 84 | 3.741398 | 6 | h | 2.032 | 3.888 | 1.28 |
| 20160807_ | SLS | 119 | 4.178427 | 9 | t | 2.432 | 4.384 | 1.4 |
| 20160807_ | SLS | 119 | 4.178427 | 9 | f | 2.432 | 4.384 | 1.4 |
| 20160807_ | SLS | 119 | 4.178427 | 9 | h | 2.432 | 4.384 | 1.4 |
| 20160807_ | SLS | 121 | 4.257886 | 3 | f | 2.304 | 4.608 | 1.288 |
| 20160807_ | SLS | 121 | 4.257886 | 3 | h | 2.304 | 4.608 | 1.288 |
| D3TBoneV | SLS | 121 | 4.257886 | 3 | t | 2.304 | 4.608 | 1.288 |
| 20160807_ | SLS | 253 | 9.502225 | 1 | h | 2.84724 | 5.012928 | 1.59372 |
| 20160807_ | SLS | 253 | 9.502225 | 1 | f | 2.84724 | 5.012928 | 1.59372 |
| D1TBoneV | SLS | 253 | 9.502225 | 1 | t | 2.84724 | 5.012928 | 1.59372 |
| 20160807_ | SLS | 392 | 15.02467 | 21 | h | 4.4 | 7.1 | 2.9 |
| 20160807_ | SLS | 392 | 15.02467 | 21 | t | 4.4 | 7.1 | 2.9 |
| 20160807_ | SLS | 392 | 15.02467 | 21 | f | 4.4 | 7.1 | 2.9 |
| 20160807_ | SLS | 401 | 15.38224 | 14 | h | 4.1 | 6.7 | 3.4 |
| 20160807_ | SLS | 401 | 15.38224 | 14 | t | 4.1 | 6.7 | 3.4 |
| 20160807_ | SLS | 401 | 15.38224 | 14 | f | 4.1 | 6.7 | 3.4 |
| 20160807_ | SLS | 469 | 18.08387 | 10 | h | 4.3 | 7.5 | 3.2 |
| 20160807_ | SLS | 469 | 18.08387 | 10 | t | 4.3 | 7.5 | 3.2 |
| 20160807_ | SLS | 469 | 18.08387 | 10 | f | 4.3 | 7.5 | 3.2 |
| 20160807_ | SLS | 492 | 18.99766 | 11 | t | 4.2 | 7.1 | 3.1 |
| 20160807_ | SLS | 492 | 18.99766 | 11 | h | 4.2 | 7.1 | 3.1 |
| 20160807_ | SLS | 624 | 24.24131 | 12 | h | 4.5 | 7.5 | 3.7 |
| 20160807_ | SLS | 624 | 24.24131 | 12 | t | 4.5 | 7.5 | 3.7 |
| 20160807_ | SLS | 492 | 18.99766 | 11 | f | 4.2 | 7.1 | 3.1 |
| 20160807_ | SLS | 624 | 24.24131 | 12 | f | 4.5 | 7.5 | 3.7 |
| 20160807_ | SLS | 525 | 20.30874 | 20 | h | 4.4 | 7.3 | 3.1 |
| 20160807_ | SLS | 525 | 20.30874 | 20 | t | 4.4 | 7.3 | 3.1 |
| 20160807_ | SLS | 525 | 20.30874 | 20 | f | 4.4 | 7.3 | 3.1 |
| 20160807_ | SLS | 585 | 22.69253 | 22 | h | 4.6 | 7.5 | 3.4 |
| 20160807_ | SLS | 585 | 22.69253 | 22 | t | 4.6 | 7.5 | 3.4 |
| 20160807_ | SLS | 585 | 22.69253 | 22 | f | 4.6 | 7.5 | 3.4 |
| 20160807_ | SLS | 1071 | 42 | 13 | t | 6.3 | 9.8 | 7.5 |
| 20160807_ | SLS | 1071 | 42 | 13 | f | 6.3 | 9.8 | 7.5 |
| 98529000C | DLS | 1071 | 42 | 13 | h | 6.4 | 9.8 | 7.5 |
| 20160807_ | SLS | 1900 | 90 | 18 | t | 6.7 | 10.7 | 10.4 |

| | | | | | | | | |
|---|---|---|---|---|---|---|---|---|
| D18FS1_Bc | SLS | 1900 | 90 | 18 | f | 6.7 | 10.7 | 10.4 |
| 20160807_ | SLS | 1181 | 120 | 15 | h | 6.4 | 10.4 | 10.8 |
| 20160807_ | SLS | 1180 | 120 | 16 | h | 6.1 | 10 | 10.4 |
| 20160807_ | SLS | 1180 | 120 | 16 | t | 6.1 | 10 | 10.4 |
| 20160807_ | SLS | 1695 | 120 | 19 | h | 6.2 | 10.3 | 10.4 |
| D15FS1Bor | SLS | 1181 | 120 | 15 | f | 6.4 | 10.4 | 10.8 |
| D16FS1Bor | SLS | 1180 | 120 | 16 | f | 6.1 | 10 | 10.4 |
| D19FS1Bor | SLS | 1695 | 120 | 19 | f | 6.2 | 10.3 | 10.4 |

| Fwid | Twid | Hwid | Porosity | CorticalThi | CanalThick | SDthicknes | MaxThickn | LongIn |
|---|---|---|---|---|---|---|---|---|
| 0.289512 | 0.328464 | 0.13932 | 0.315417 | 186.4768 | 25.9328 | 12.7968 | 79.4224 | 0.17954 |
| 0.289512 | 0.328464 | 0.13932 | 0.362649 | 158.8608 | 34.336 | 12.7296 | 68.7072 | 0.152255 |
| 0.289512 | 0.328464 | 0.13932 | 0.222926 | 373.536 | 30.6 | 13.144 | 67.8816 | 0.430194 |
| 0.192 | 0.1944 | 0.1216 | 0.230936 | 122.24 | 27.608 | 11.064 | 65.6592 | 0.111251 |
| 0.192 | 0.1944 | 0.1216 | 0.120972 | 67.4176 | 18.9968 | 6.0048 | 31.6784 | 0.27068 |
| 0.192 | 0.1944 | 0.1216 | 0.329008 | 328.5504 | 27.3744 | 13.2064 | 68.3328 | 0.493126 |
| 0.192 | 0.1944 | 0.1216 | 0.305252 | 336.9664 | 21.808 | 7.2848 | 46.592 | 0.545656 |
| 0.192 | 0.1944 | 0.1216 | 0.383442 | 342.1376 | 31.2416 | 14.248 | 86.9312 | 0.285249 |
| 0.192 | 0.1944 | 0.1216 | 0.154941 | 103.7184 | 22.2928 | 10.4544 | 48.8464 | 0.386642 |
| 0.1976 | 0.2152 | 0.1176 | 0.354922 | 417.5808 | 32.8208 | 11.8368 | 73.6 | 0.17509 |
| 0.338616 | 0.384984 | 0.151776 | 0.470907 | 264.5696 | 48.5104 | 19.8032 | 91.7456 | 0.210418 |
| 0.1976 | 0.2152 | 0.1176 | 0.394488 | 439.552 | 34.3264 | 12.7952 | 67.9584 | 0.427046 |
| 0.338616 | 0.384984 | 0.151776 | 0.398573 | 445.2928 | 35.7168 | 14.7312 | 72.76 | 0.318999 |
| 0.338616 | 0.384984 | 0.151776 | 0.401494 | 427.0272 | 35.2608 | 14.2848 | 82.1472 | 0.115811 |
| 0.1976 | 0.2152 | 0.1176 | 0.193351 | 104.6912 | 19.272 | 6.2304 | 35.0544 | 0.065334 |
| 0.2416 | 0.3 | 0.1416 | 0.440936 | 496.48 | 35.9264 | 14.0992 | 73.8784 | 0.322328 |
| 0.2416 | 0.3 | 0.1416 | 0.342142 | 368.1984 | 26.3552 | 10.1248 | 57.3328 | 0.149392 |
| 0.2416 | 0.3 | 0.1416 | 0.413495 | 200.9792 | 37.7776 | 14.3104 | 69.0784 | 0.185537 |
| 0.312 | 0.236 | 0.1544 | 0.402113 | 469.2032 | 32.6784 | 12.5232 | 74.9792 | 0.131604 |
| 0.312 | 0.236 | 0.1544 | 0.360208 | 141.088 | 31.552 | 13.896 | 63.84 | 0.133225 |
| 0.312 | 0.236 | 0.1544 | 0.33703 | 511.0464 | 35.3888 | 13.1744 | 78.384 | 0.493126 |
| 0.330336 | 0.490752 | 0.215496 | 0.364114 | 292.896 | 31.6208 | 11.048 | 68.184 | 0.27329 |
| 0.330336 | 0.490752 | 0.215496 | 0.383769 | 880.2048 | 36.328 | 14.8352 | 76.1312 | 0.140159 |
| 0.330336 | 0.490752 | 0.215496 | 0.266346 | 589.1392 | 34.6672 | 18.04 | 107.5696 | 0.445037 |
| 0.4 | 0.4 | 0.3 | 0.333855 | 243.9232 | 46.1072 | 29.5712 | 115.9088 | 0.335159 |
| 0.4 | 0.4 | 0.3 | 0.373339 | 839.4368 | 42.2672 | 15.0848 | 81.8352 | 0.476915 |
| 0.4 | 0.4 | 0.3 | 0.26288 | 587.7184 | 31.088 | 14.0816 | 72.2656 | 0.223535 |
| 0.4 | 0.3 | 0.2 | 0.448151 | 338.7776 | 34.984 | 12.2896 | 77.3984 | 0.179739 |
| 0.4 | 0.3 | 0.2 | 0.380942 | 869.4464 | 32.5264 | 13.7776 | 100.6352 | 0.303544 |
| 0.4 | 0.3 | 0.2 | 0.269659 | 628.672 | 24.4752 | 9.5344 | 74.7728 | 0.160947 |
| 0.4 | 0.4 | 0.2 | 0.327937 | 323.5776 | 29.32 | 11.2384 | 74.704 | 0.32095 |
| 0.4 | 0.4 | 0.2 | 0.351417 | 805.7856 | 40.8976 | 15.2256 | 111.6336 | 0.342908 |
| 0.4 | 0.4 | 0.2 | 0.225679 | 675.072 | 25.4384 | 9.7312 | 65.9696 | 0.1459 |
| 0.3 | 0.4 | 0.3 | 0.312676 | 725.9968 | 39.56 | 27.9168 | 107.9504 | 0.627462 |
| 0.3 | 0.4 | 0.3 | 0.329429 | 251.2832 | 30.5328 | 12.1584 | 60.7152 | 0.265938 |
| 0.5 | 0.4 | 0.3 | 0.354353 | 403.3216 | 32.7696 | 17.0128 | 100.8896 | 0.24777 |
| 0.5 | 0.4 | 0.3 | 0.350048 | 1035.744 | 44.3696 | 19.2304 | 109.4096 | 0.403148 |
| 0.3 | 0.4 | 0.3 | 0.230171 | 561.1584 | 27.3536 | 13.3184 | 75.8608 | 0.138525 |
| 0.5 | 0.4 | 0.3 | 0.343908 | 1011.462 | 37.6816 | 15.184 | 86.9312 | 0.159553 |
| 0.4 | 0.4 | 0.3 | 0.349077 | 208.1024 | 26.4512 | 10.6512 | 65.8928 | 0.177743 |
| 0.4 | 0.4 | 0.3 | 0.324138 | 889.4912 | 30.952 | 10.5888 | 63.84 | 0.413321 |
| 0.4 | 0.4 | 0.3 | 0.326495 | 787.6288 | 31.5504 | 11.8784 | 98.5792 | 0.171153 |
| 0.5 | 0.4 | 0.3 | 0.376322 | 405.0304 | 35.4448 | 13.7072 | 84.9664 | 0.155724 |
| 0.5 | 0.4 | 0.3 | 0.344801 | 1025.536 | 42.136 | 16.9904 | 86.1024 | 0.402995 |
| 0.5 | 0.4 | 0.3 | 0.254005 | 709.2224 | 28.216 | 12.1824 | 76.6 | 0.122619 |
| 0.6 | 0.6 | 0.6 | 0.101099 | 593.7856 | 24.1904 | 15.6704 | 86.4592 | 0.274505 |
| 0.6 | 0.6 | 0.6 | 0.160151 | 705.8688 | 26.6032 | 13.408 | 68.3328 | 0.08997 |
| 0.6 | 0.6 | 0.6 | 0.434347 | 795.8144 | 18.696 | 9.9728 | 65.6592 | 0.292258 |
| 0.7 | 0.6 | 0.85 | 0.04367 | 681.28 | 14.064 | 4.1056 | 27.3408 | 0.489202 |

| | | | | | | | | | |
|---|---|---|---|---|---|---|---|---|---|
| 0.7 | 0.6 | 0.85 | 0.031121 | 490.048 | 10.1344 | 3.4592 | 27.7136 | 0.361861 |
| 0.7 | 0.7 | 0.9 | 0.014102 | 755.3984 | 7.4176 | 2.7296 | 25.6 | 0.168839 |
| 0.6 | 0.7 | 0.9 | 0.011269 | 683.6032 | 8.9408 | 3.6576 | 27.528 | 0.386107 |
| 0.6 | 0.7 | 0.9 | 0.018228 | 766.6688 | 15.0528 | 10.16 | 49.6768 | 0.377336 |
| 0.8 | 0.7 | 0.9 | 0.029602 | 748.1408 | 13.1968 | 10.8064 | 68.0336 | 0.176765 |
| 0.7 | 0.7 | 0.9 | 0.017514 | 592.5632 | 13.4416 | 8.4944 | 46.7024 | 0.25068 |
| 0.6 | 0.7 | 0.9 | 0.030531 | 784.3456 | 25.6592 | 21.9312 | 88.2752 | 0.188192 |
| 0.8 | 0.7 | 0.9 | 0.03442 | 527.584 | 24.0352 | 18.3648 | 76.3312 | 0.277278 |

| CircIn | Radin | OblIn | SecondMoment |
|---|---|---|---|
| 0.25228 | 0.200795 | 0.367386 | 1.462156 |
| 0.26668 | 0.085734 | 0.495332 | 0.119266 |
| 0.227784 | 0.082411 | 0.259611 | 3.67839 |
| 0.179288 | 0.351099 | 0.358362 | 0.280202 |
| 0.224186 | 0.151709 | 0.353425 | 0.040254 |
| 0.216197 | 0.073569 | 0.217108 | 0.566428 |
| 0.152871 | 0.078574 | 0.222898 | 0.573334 |
| 0.220559 | 0.162857 | 0.331335 | 0.552614 |
| 0.184852 | 0.117612 | 0.310894 | 0.056535 |
| 0.180772 | 0.229453 | 0.414685 | 0.665221 |
| 0.170924 | 0.231261 | 0.387398 | 0.213594 |
| 0.079342 | 0.064424 | 0.429188 | 0.923916 |
| 0.224336 | 0.133193 | 0.323471 | 7.018596 |
| 0.199754 | 0.249365 | 0.435069 | 4.435678 |
| 0.149424 | 0.276363 | 0.508879 | 0.051032 |
| 0.188573 | 0.160191 | 0.328908 | 3.179563 |
| 0.152905 | 0.232493 | 0.46521 | 1.281809 |
| 0.16851 | 0.236338 | 0.409615 | 0.14544 |
| 0.150599 | 0.155755 | 0.562042 | 3.53955 |
| 0.253378 | 0.030601 | 0.582795 | 0.15453 |
| 0.216197 | 0.073569 | 0.217108 | 1.365432 |
| 0.315286 | 0.090683 | 0.320741 | 0.760977 |
| 0.187463 | 0.244483 | 0.427896 | 5.566908 |
| 0.242977 | 0.078528 | 0.233458 | 18.97796 |
| 0.243903 | 0.115402 | 0.305536 | 2.021043 |
| 0.135747 | 0.087987 | 0.299351 | 11.14153 |
| 0.321638 | 0.10049 | 0.354337 | 9.44191 |
| 0.317606 | 0.149848 | 0.352807 | 0.635264 |
| 0.015166 | 0.025792 | 0.655498 | 3.851919 |
| 0.247253 | 0.143332 | 0.448468 | 9.788863 |
| 0.333177 | 0.06652 | 0.279353 | 0.620975 |
| 0.098742 | 0.055891 | 0.502459 | 10.96895 |
| 0.293859 | 0.14152 | 0.418722 | 10.14613 |
| 0.157798 | 0.045606 | 0.169133 | 10.49732 |
| 0.41407 | 0.081178 | 0.238814 | 2.066464 |
| 0.409945 | 0.070757 | 0.271529 | 2.840003 |
| 0.060125 | 0.053945 | 0.482782 | 11.88739 |
| 0.269662 | 0.171104 | 0.420709 | 3.365896 |
| 0.230326 | 0.175517 | 0.434604 | 26.82369 |
| 0.404023 | 0.124235 | 0.293999 | 1.788305 |
| 0.013912 | 0.066339 | 0.506427 | 11.37189 |
| 0.295493 | 0.137824 | 0.39553 | 10.86956 |
| 0.30776 | 0.128637 | 0.407879 | 2.847067 |
| 0.055964 | 0.035283 | 0.505759 | 11.85818 |
| 0.349504 | 0.089821 | 0.438055 | 22.6025 |
| 0.182951 | 0.173629 | 0.368915 | 37.28869 |
| 0.169582 | 0.269561 | 0.470887 | 41.86206 |
| 0.400443 | 0.056655 | 0.250644 | 45.07841 |
| 0.218938 | 0.046071 | 0.245789 | 40.91426 |

| | | | |
|---|---|---|---|
| 0.230473 | 0.13654 | 0.271127 | 53.39319 |
| 0.434127 | 0.105389 | 0.291645 | 167.6392 |
| 0.306506 | 0.088341 | 0.219046 | 155.4512 |
| 0.239123 | 0.106432 | 0.27711 | 74.01999 |
| 0.212598 | 0.137368 | 0.47327 | 166.4385 |
| 0.276528 | 0.156256 | 0.316536 | 61.73709 |
| 0.393103 | 0.179866 | 0.238839 | 44.68955 |
| 0.381145 | 0.05558 | 0.285997 | 86.87487 |

| Scan | Mass | Age | Age (days) | Animal | Bone | Flen | Tlen | Hlen |
|---|---|---|---|---|---|---|---|---|
| 98498Bone | 67.5 | 2 | 14 | a | t | 4.4 | 3.4 | 3.2 |
| 984990014 | 67.5 | 2 | 14 | a | h | 4.4 | 3.4 | 3.2 |
| 985020008 | 67.5 | 2 | 14 | a | f | 4.4 | 3.4 | 3.2 |
| 985070028 | 67 | 2 | 14 | b | f | 4.4 | 3.4 | 3.2 |
| 98508Bone | 67 | 2 | 14 | b | t | 4.4 | 3.4 | 3.2 |
| 985090008 | 67 | 2 | 14 | b | h | 4.4 | 3.4 | 3.2 |
| 98542Bone | 67.6 | 2 | 14 | c | h | 4.3 | 3.3 | 3.2 |
| 98559Bone | 67.6 | 2 | 14 | c | f | 4.3 | 3.3 | 3.2 |
| 98565Bone | 67.6 | 2 | 14 | c | t | 4.3 | 3.3 | 3.2 |
| 98501Bone | 90 | 3 | 21 | a | f | 5.4 | 4.1 | 4 |
| 985050019 | 90 | 3 | 21 | a | h | 5.4 | 4.1 | 4 |
| 985060045 | 90 | 3 | 21 | a | t | 5.4 | 4.1 | 4 |
| 984770012 | 250 | 5 | 35 | a | t | 7 | 5.6 | 5.3 |
| 984840017 | 250 | 5 | 35 | a | f | 7 | 5.6 | 5.3 |
| 984850006 | 250 | 5 | 35 | a | h | 7 | 5.6 | 5.3 |
| 985100002 | 300 | 5 | 35 | b | h | 7.7 | 5.8 | 5.4 |
| 985180011 | 300 | 5 | 35 | b | f | 7.7 | 5.8 | 5.4 |
| 985430009 | 269 | 5 | 35 | c | t | 7.4 | 5.3 | 5.4 |
| 985440011 | 269 | 5 | 35 | c | f | 7.4 | 5.3 | 5.4 |
| 985460017 | 269 | 5 | 35 | c | h | 7.4 | 5.3 | 5.4 |
| 985500005 | 269 | 5 | 35 | b | t | 7.7 | 5.8 | 5.4 |
| 984820015 | 204.8 | 6 | 42 | a | f | 7.4 | 5.4 | 5.3 |
| 984830015 | 204.8 | 6 | 42 | a | t | 7.4 | 5.4 | 5.3 |
| 98488Bone | 204.8 | 6 | 42 | a | h | 7.4 | 5.4 | 5.3 |
| 985190001 | 292.6 | 6 | 42 | b | t | 7.3 | 5.5 | 5.4 |
| 985470011 | 306.7 | 6 | 42 | c | t | 7.4 | 5.8 | 5.5 |
| 985480028 | 306.7 | 6 | 42 | c | h | 7.4 | 5.8 | 5.5 |
| 985490028 | 306.7 | 6 | 42 | c | f | 7.3 | 5.5 | 5.4 |
| 985670013 | 292.6 | 6 | 42 | b | f | 7.4 | 5.8 | 5.5 |

| Fwid | Twid | Hwid | Porosity | CorticalThi | CanalThick | SDthicknes | MaxThickn | LongIn |
|---|---|---|---|---|---|---|---|---|
| 0.27 | 0.25 | 0.3 | 0.197549 | 422.5344 | 19.8432 | 6.5904 | 45.2544 | 0.298049 |
| 0.27 | 0.25 | 0.3 | 0.194946 | 321.472 | 18.9104 | 7.056 | 51.2992 | 0.331106 |
| 0.27 | 0.25 | 0.3 | 0.12747 | 249.3504 | 15.5232 | 6.1152 | 38.6656 | 0.158198 |
| 0.25 | 0.2 | 0.3 | 0.170598 | 277.8752 | 6.4 | 0 | 6.4 | 0.224202 |
| 0.25 | 0.2 | 0.3 | 0.236203 | 345.0944 | 22.9664 | 9.232 | 56.0688 | 0.455752 |
| 0.25 | 0.2 | 0.3 | 0.243887 | 318.0544 | 24.4432 | 8.3424 | 51.4 | 0.183145 |
| 0.2 | 0.2 | 0.25 | 0.254502 | 360.736 | 22.2016 | 6.6096 | 45.8176 | 0.108745 |
| 0.2 | 0.2 | 0.25 | 0.134425 | 335.36 | 15.5072 | 5.5776 | 34.4656 | 0.420093 |
| 0.2 | 0.2 | 0.25 | 0.188068 | 421.5488 | 18.728 | 6.6352 | 48.9504 | 0.578058 |
| 0.3 | 0.3 | 0.4 | 0.343384 | 757.9712 | 37.8944 | 11.4608 | 49.8832 | 0.118906 |
| 0.3 | 0.3 | 0.4 | 0.271151 | 1102.464 | 30.1744 | 11.4656 | 76.0624 | 0.040145 |
| 0.3 | 0.3 | 0.4 | 0.320059 | 780.7104 | 37.2288 | 14.5184 | 79.0336 | 0.158022 |
| 0.4 | 0.45 | 0.5 | 0.188375 | 681.536 | 29.6448 | 15.5936 | 75.5904 | 0.319585 |
| 0.4 | 0.45 | 0.5 | 0.130759 | 461.44 | 22.984 | 12.1088 | 61.5536 | 0.285123 |
| 0.4 | 0.45 | 0.5 | 0.099368 | 411.072 | 22.1312 | 14.3504 | 77.5296 | 0.371671 |
| 0.5 | 0.4 | 0.5 | 0.293897 | 906.6496 | 33.7152 | 16.344 | 107.1408 | 0.070948 |
| 0.5 | 0.4 | 0.5 | 0.110866 | 430.3296 | 13.0208 | 4.3088 | 35.7776 | 0.205086 |
| 0.4 | 0.4 | 0.5 | 0.14099 | 518.6112 | 24.1248 | 11.9408 | 56.7936 | 0.508253 |
| 0.4 | 0.4 | 0.5 | 0.251989 | 776.5632 | | | | 0.122144 |
| 0.4 | 0.4 | 0.5 | 0.206254 | 581.9072 | 31.8064 | 14.7936 | 71.3392 | 0.083818 |
| 0.5 | 0.4 | 0.5 | 0.179 | 754.4192 | 27.472 | 15.4912 | 75.928 | 0.205884 |
| 0.4 | 0.4 | 0.45 | 0.048667 | 263.7184 | 9.3264 | 2.4416 | 22.6272 | 0.10338 |
| 0.4 | 0.4 | 0.45 | 0.063155 | 436.8 | 13.624 | 3.9072 | 28.8 | 0.426625 |
| 0.4 | 0.4 | 0.45 | 0.017861 | 560.4992 | 8.52 | 2.1136 | 13.1936 | 0.313506 |
| 0.4 | 0.4 | 0.5 | 0.134235 | 531.7184 | 25.4848 | 13.584 | 64.8736 | 0.332635 |
| 0.4 | 0.4 | 0.4 | 0.122606 | 593.4848 | 25.7216 | 14.904 | 68.4832 | 0.254234 |
| 0.4 | 0.4 | 0.4 | 0.17844 | 403.104 | 26.7952 | 13.0304 | 67.504 | 0.302669 |
| 0.4 | 0.4 | 0.5 | 0.214203 | 477.6704 | 29.8176 | 18.1056 | 100.3808 | 0.270137 |
| 0.4 | 0.4 | 0.4 | 0.120452 | 367.04 | 22.376 | 10.6336 | 52.1904 | 0.210658 |

| CircIn | Radin | Oblin | SecondMoment |
|---|---|---|---|
| 0.235989 | 0.142206 | 0.323755 | 1.549271 |
| 0.326295 | 0.058067 | 0.284532 | 2.46095 |
| 0.248215 | 0.165571 | 0.428015 | 1.456085 |
| 0.19083 | 0.18378 | 0.401188 | 1.216056 |
| 0.198142 | 0.07924 | 0.266865 | 0.640919 |
| 0.261092 | 0.148938 | 0.406825 | 2.443298 |
| 0.17503 | 0.280081 | 0.436144 | 1.426328 |
| 0.169625 | 0.076907 | 0.333375 | 0.632136 |
| 0.182352 | 0.04967 | 0.189921 | 0.697464 |
| 0.243981 | 0.186828 | 0.450285 | 3.737971 |
| 0.25697 | 0.181964 | 0.520921 | 12.05669 |
| 0.159284 | 0.286799 | 0.395895 | 3.765843 |
| 0.255444 | 0.11225 | 0.31272 | 15.37567 |
| 0.250809 | 0.11536 | 0.348708 | 8.165408 |
| 0.231207 | 0.11258 | 0.284542 | 15.72471 |
| 0.179735 | 0.284457 | 0.464859 | 25.61747 |
| 0.424887 | 0.063899 | 0.306128 | 16.2686 |
| 0.258147 | 0.035395 | 0.198206 | 8.783982 |
| 0.279778 | 0.177813 | 0.420265 | 10.80676 |
| 0.37622 | 0.130045 | 0.409917 | 20.04876 |
| 0.256667 | 0.155887 | 0.381562 | 10.67587 |
| 0.430632 | 0.087829 | 0.378159 | 5.428469 |
| 0.195331 | 0.092511 | 0.285534 | 7.876638 |
| 0.249604 | 0.147661 | 0.289229 | 13.72973 |
| 0.239684 | 0.10688 | 0.320801 | 8.916081 |
| 0.33354 | 0.093822 | 0.318404 | 9.492627 |
| 0.37118 | 0.073365 | 0.252786 | 7.459011 |
| 0.323114 | 0.107356 | 0.299392 | 8.348195 |
| 0.301365 | 0.152534 | 0.335442 | 6.981771 |
